# Meta-Analysis and Machine Learning Models to Optimize the Efficiency of Self-Healing Capacity of Cementitious Material

**DOI:** 10.3390/ma14164437

**Published:** 2021-08-08

**Authors:** Shashank Gupta, Salam Al-Obaidi, Liberato Ferrara

**Affiliations:** 1 Department of Civil and Environmental Engineering, Politecnico di Milano, 20133 Milan, Italy; salammaytham.alobaidi@polimi.it (S.A.-O.); liberato.ferrara@polimi.it (L.F.); 2 Roads and Transportations Engineering Department, University of Al-Qadisiyah, Diwaniyah 58001, Iraq

**Keywords:** artificial neural network, self-healing concrete, meta-analysis, systematic review

## Abstract

Concrete and cement-based materials inherently possess an autogenous self-healing capacity. Despite the huge amount of literature on the topic, self-healing concepts still fail to consistently enter design strategies able to effectively quantify their benefits on structural performance. This study aims to develop quantitative relationships through statistical models and artificial neural network (ANN) by establishing a correlation between the mix proportions, exposure type and time, and width of the initial crack against suitably defined self-healing indices (SHI), quantifying the recovery of material performance. Furthermore, it is intended to pave the way towards consistent incorporation of self-healing concepts into durability-based design approaches for reinforced concrete structures, aimed at quantifying, with reliable confidence, the benefits in terms of slower degradation of the structural performance and extension of the service lifespan. It has been observed that the exposure type, crack width and presence of healing stimulators such as crystalline admixtures has the most significant effect on enhancing SHI and hence self-healing efficiency. However, other parameters, such as the amount of fibers and Supplementary Cementitious Materials have less impact on the autogenous self-healing. The study proposes, through suitably built design charts and ANN analysis, a straightforward input–output model to quickly predict and evaluate, and hence “design”, the self-healing efficiency of cement-based materials.

## 1. Introduction

Cementitious materials undergo the natural process of autogenous healing that is total or partial self-closure of cracks. The closure of these cracks subsequently results in partial regaining of concrete initial strength as well as improving the durability performance. In 1973, tests were performed on cement mortars used for the lining of iron water pipes, to study the autogenous self-healing under the pipe service water as well as non-service water conditions [1]. The study reported substantial self-healing in both cases, whereas the latter mentioned exposure condition showed a slightly better rate of healing. Correspondingly, several old structures, bridges, as well as buildings have shown exceptional durability, resulting in unpredicted longevity [2,3]. This behavior has been contributed to by the autogenous self-healing property of concrete.

### 1.1. Mechanism of Autogenous Self-Healing of Concrete

Several self-healing mechanisms and their efficacy have been investigated to promote the self-healing properties of concrete. These mechanisms include are continuing hydration, dissolution and crystallization, particle clogging, and carbonation [4]. The process of continuing hydration in autogenous healing is a principal step in recovering the mechanical properties as the newly formed hydrated products have similar strength properties as the calcium silicate hydrate (CSH) gel. The microstructural characterization of healed concrete through SEM-EDX analysis shows the presence of CSH, ettringite, and calcium hydroxide (Ca(OH)_2_) in healed cracks [5]. Based on the mechanism of autogenous self-healing of cementitious material, several parameters have been determined that influence the rate of self-healing. Crack width, mix design, fiber content, together with treatment duration, and environmental exposure conditions are the most significant variables that affect the self-healing of concrete [6].

### 1.2. Crack Width

The autogenous healing is more effective when the cracks are smaller. As a result, the autogenous, inherent healing capability of cement-based materials may be significantly enhanced by restricting and regulating the fracture width. In this respect, many studies have been done to determine the relationship between performance recovery rate and initial crack width for different types of concrete. For high performance concrete (HPC) with fly ash, micro-silica, and water-reducing agent, a logarithmic relation was obtained between the normalized flow rate recovery and time of exposure. It was observed that for larger cracks, the permeability was increasing at a higher rate. The experimental results were also in close agreement with the law of Hagen–Poiseuille [7]. Furthermore, cracks can function as water storage as well as carriers across the binder matrix, collecting water during wet phases of wet/dry loops and expelling it during dry times, therefore triggering the ongoing hydration as well as carbonation processes essential for crack healing [6].

### 1.3. Admixture

The addition of supplementary cementitious materials (SCM) and crystalline admixture (CA) can additionally improve the rate and efficiency of self-healing as a “stimulated-autogenous healing” approach. The partial substitution of cement with fly ash along with the addition of crystalline admixture can lead to a recovery of both compressive strength and electrical resistivity of concrete up to 94% [8]. It was observed that after 30 days of water curing, a sample containing a high volume of fly ash regained 74% of the loss of the compressive strength, whereas samples without fly ash regained only 68%. Furthermore, more pronounced improvements were observed in permeability analysis [9]. Similarly, a crack healing rate of around 81% to 93% was reported for concrete containing CA. On the other hand, the specimens without CA exhibited a crack healing rate of 80–86%. Cracks with a width up 0.25 mm were healed in the specimens containing CA [10]. The attempts have been further made to review the mechanisms of healing stimulation by crystalline admixtures [11].

### 1.4. Fibers and Polymers

Polymers are widely used to enhance the self-healing of concrete either by encapsulation method, vascular method, or by directly injecting the polymers into the crack. Some of these polymers are polyurethane, superabsorbent polymers (SAP), acrylamide, acrylate, epoxy, poly styrene-divinylbenzene, styrene-butadiene rubber, etc. The inclusion of these polymers improves the regain ratio of mechanical properties and increases the surface crack healing [12].

Numerous experiments, as well as studies, have also been conducted on the concrete reinforced with synthetic fibers and steel fibers. The fibers control the opening of the cracks that make self-healing products to easily attach to the surface of the cracks and fill them. Permeability tests and microstructural characterization of the engineered cementitious composite (ECC) containing different synthetic fibers, like polypropylene (PP), ethylene vinyl alcohol (EVA), and poly-vinyl acrylate (PVA), have been performed. The study concluded that the precipitation of calcium carbonate potentially increases with the inclusion of synthetic fibers [13], fibers acting, thanks to the nature of their surface, as nucleation sites for the precipitation of crack healing products. A similar result was obtained by a study dealing with concrete containing PVA microfibers and SAP [14]. 

### 1.5. Treatment Environment and Period of Exposure

The environment and the time of exposure have been varied by different researchers to either optimize the treatment condition for concrete under investigation or to investigate the effect of the intended field of investigation. Some of these treatment conditions are tap/distilled water curing at a different temperature, air chambers with controlled relative humidity (RH) and temperature, wet/dry cycle, freeze/thaw cycles, etc. [15]. In the case of ECCs, the mechanical regain was also investigated, resulting, e.g., after 90 days of water immersion, in a 60% and 70% regain of stiffness and tensile strength, respectively [16]. The results always showed that the presence of water or high humidity is essential for the rapid and effective healing of the concrete. In addition to the conditioning environment, the duration of treatment is also an essential parameter, continuation of the healing capacity up to longer ages, though with slower rate, having been documented [15].

Similarly, ultra high-performance (or fiber-reinforced) concretes (UHPC/UHPFRC) materials, due to their mix composition, featuring a high binder content and a low water/binder ratio [17,18,19], have also been identified as highly conducive to exhibit autogenous self-healing capacity, also thanks to their ability to spread and control the damage through small crack width, which works as an excellent governing parameter for self-healing in the presence of water, promoting the delayed hydration of the available substantial amounts of unhydrated binder, and with the presence of the seal healing promoters as well [20,21,22,23,24,25,26]. 

### 1.6. Systematic Review and Meta-Analysis

It is evident from the literature that several factors influence the efficiency of self-healing, which makes the systematic review of research the best supporting evidence. However, this form of review must be supported by statistical mathematical tools like meta-data analysis. This form of analysis is quite common in evidence-based healthcare research. Multiple clinical trials are performed by different labs, and the results are publicly filed. The systematic reviews summarize the results of different labs with several statistical parameters. The first systematic review was performed in 1747 by James Lind to find the optimum treatment of scurvy [27].

In the field of cement-based construction materials, a few systematic reviews have been performed. Moreover, meta-data analyses are rarer in the same field. A recent systematic review has been done to study the effect of fly-ash replacement in high volume fly-ash concrete (HVFAC) [28]. In this study, the mechanical and durability properties of HVFAC of different mix designs were numerically compared. However, the study was limited to the numerical presentation of the values of flexural strength, compressive strength, splitting tensile strength, and elasticity modulus. The meta-data analysis was not carried out. Similarly, the effects of recycled fibers of different types on the mechanical, durability, and physical properties of concrete have been investigated using a systematic review. The effect of plastic, metal, and natural fibers on the performance of different cementitious matrices was analyzed. The effect of variables on self-healing was qualitatively compared, but the correlation of data was not developed by any mathematical model [29].

A meta-analysis study has been done, where the parameters of mix-design of self-consolidating concrete have been related to fresh state parameters of concrete [30]. In this study, the meta-analysis has been performed by determining the collected variables as input and output data. The amount of cement, water content, amount of SCM, and aggregate size were taken as input data. On the other hand, the results from slump-flow tests were taken as output data. The multiple linear regression was performed to study the impact of input variables on the output variables.

### 1.7. Artificial Neural Network (ANN) Model

On a separate note, new predictive models are being developed to determine the properties of concrete in the rapidly evolving concrete industry. The existing models for predicting the durability and mechanical properties of different types of concrete are usually based on empirical formulations. These formulations are obtained through linear or nonlinear best fitting models developed from the experimental data. In recent studies, neural networks have been used extensively for predicting the durability and mechanical parameters of the cement-based systems, also exploiting the extensive datasets created by researchers, based on the experimental data or the existing literature review. For instance, the durability of RC concrete was predicted using ANN models by analyzing the deterioration of the concrete samples using accelerated chloride tests. The researchers used around 250 points to analyze the influence of cement type, period of exposure, amount of SCM, and the w/b ratio on the chloride penetration in the concrete specimens [31].

In a separate study, the compressive strength of geopolymer concrete has been predicted based on the mix design and the age of specimen. The 70:15:15 ratio of total data has been used for training, validation, and testing of the model. The total number of data points considered in the study were 117. The correlation coefficient, R, was lying in the range of 0.95–0.96. The results were also compared with the Gene Expression Programming (GEP) model, another type of computational model. It was shown that ANN model performed better than the classic regression model [32].

### 1.8. Study Objective

A substantial study and review have been carried out on autogenous healing of different cement-based materials. The construction industry is interested in concrete that does not decay; however, further research is needed to perform the standardization and codification of the existing findings. The contractors and owners globally, depend on the local national codes for infrastructure construction. With the present-day understanding of the self-healing of concrete, a huge proportion of these contractors may not take the risk of promising repair-free concrete. Therefore, more quantitative proofs, depending on multiple studies, correlating the several involved factors to the self-healing of concrete must be provided.

One way of supporting this activity is to carry out a state-of-the-art quantitative study that unifies the observations of numerous studies on an individual scale through data-mining and statistical models. This study will lay down a comparison and present in a quantitative way the effect of numerous parameters on the autogenous self-healing of cementitious material. The first objective is to determine the factors affecting the autogenous self-healing of concrete by analyzing multiple existing literatures. It is then followed by developing a meta-data statistical model determining the impact as well as the reliability of these factors on the self-healing property. Finally, an ANN model is to develop to present a robust simulation model to predict the self-healing capacity of different cementitious materials on a uniform scale. 

## 2. Methodology

**Figure 1:**
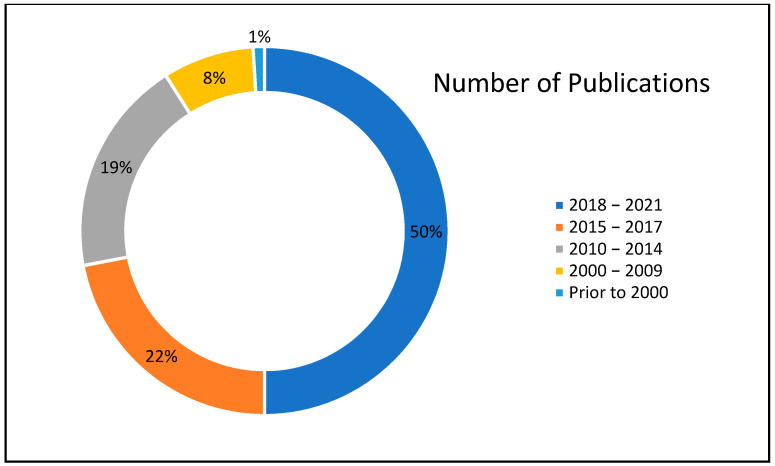
Distribution of timeline of publication of relevant studies.

The relevant data has been collected from numerous peer-reviewed articles. “ScienceDirect” was a major scientific database to collect the relevant articles, journals, and book chapters. In the initial survey, 236 papers related to the self-healing capacity of concrete were analyzed. However, based on the required data, several papers were later removed because of the inadequacy or incompleteness of the data provided. The total number of relevant papers collected for both meta-analysis is 100. The distribution of the timeline of publications is presented in Figure 1. It can be observed that half of the papers have been published between 2018 and 2021. The total number of data points obtained from the collected literature was 2786.

The input parameters collected from each of these studies are the dosage of cement (kg/m^3^) and of supplementary cementitious materials (SCM) (kg/m^3^), fiber type and content (%vol), water/cement ratio, crack width (μm), age of treatment (days), and self-healing environment. The parameters collected as output are relevant results of the self-healing characterization test and geometrical closure of cracks. The relevant results are then converted into “Self-Healing Indices” (SHI) to maintain the consistency of results. These parameters for different tests are determined according to the experiment and the initial data. The values are being normalized to 1.0 by simple mathematical manipulations. The formulas of self-healing indices for several experiments are presented in Table 1.

The self-healing indices have then been used to develop forest plots. A forest plot is a graph that displays results from several studies. The inference of the forest plot is a diamond that represents the result of a meta-analysis of the considered studies. The position of the diamond represents Cohen’s *d*-value (effect size), and its width represents the 95% confidence interval (CI). The mean and standard deviations of SHI values are used to calculate the margin of error (*MoE*) (Equation (1)) and Cohen’s *d*-value (Equations (2) and (3)) [33]. The Cohen’s *d*-value is used to represent the magnitude of the effect of the certain parameter under analysis. The meta-analysis results of the various comparison will be shown in terms of Cohen’s *d*-value and *MoE*.

(1) $$MoE=0.5*\left(SHI_{mean}+z\frac{SHI_{sd}}{\sqrt{N}}\right)$$

where, $SHI_{mean}$ is mean, $SHI_{sd}$ is the standard deviation, *z* is *Z*-value (i.e., 1.96 for 95% CI), and *N* is the sample size.

(2) $$d=\left(SHI_{1,mean}-SHI_{2,mean}\right)/SHI_{sd,p})$$

where ‘d’ is Cohen’s *d*, $SHI_{1,mean}$ and $SHI_{2,mean}$ are mean of SHI of two data-sets, and $SHI_{sd,p}$ is pooled standard deviation.

(3) $$SHI_{sd,p}=\sqrt{\frac{\left(N_{1}-1\right)SHI_{1,sd}{}^{2}+\left(N_{2}-1\right)SHI_{2,sd}{}^{2}}{\left(N_{1}-1\right)+\left(N_{2}-1\right)}}$$

where, $SHI_{1,sd}$ and $SHI_{2,sd}$ are standard deviations of two data-sets and $N_{1}$ and $N_{2}$ are a sample size of two data sets. 

The relative importance analysis has been carried out from the collected dataset to calculate the importance percentage of total cementitious material, water to cement content, crack width, age of healing, and fiber content in determining the self-healing index of concrete. Tonidandel and LeBreton’s method has been used to calculate the relative weight percentage [34]. The technique is an alternative to multi-regression analysis and considers the problem of multicollinearity. This analysis breaks down the overall variance projected by a regression model into the weights that correctly reflect the proportionate impact of each predictor component. The method helps to lay out the importance rank of the variable based on their contribution to R-squared [35]. The analysis was performed in R using the open-source ‘rwa()’ package developed by Tonidandel [34]. The input parameters considered were the fiber content (%vol), age of healing (days), crack width (μm), *w/c*, and total cementitious material (kg/m^3^) (TCM). The relative importance analysis was carried out against the output of SHI. 

Furthermore, based on the collected data, optimization charts have been developed for optimized design concerning the self-healing capacity of cementitious material. The Delaunay triangulation has been implemented to evaluate the interpolated values of SHI to generate the contour lines. Delaunay triangulation is used as an optimal method to generate a linear interpolation function that can be used to reconstruct the functional approximation in three-dimensional space. The Delaunay triangulation is applied to the pair of chosen parameters (like cement content, crack width, etc.) and linear interpolation functions are constructed based on the value of response (SHI) [36]. The charts are prepared in MATLAB (v9.5, R2018b) using the ‘scatteredinterpolant’ function. Furthermore, to have clean contour plots with uniform gaps in SHI value, the MATLAB generated charts are traced in AutoCAD (2017) manually.

For ANN analysis, a back-propagation network has been developed in this study. A single-layer feed-forward neural network has been developed. In this network, the output and hidden layers are associated with biases. The training of data is performed by the method of supervised learning in this algorithm. The predicted output values are compared with the provided outputs and the associated error is calculated. The weights and biases of each neural network are adjusted based on this error. This process is iteratively carried out until the error is below the desired value or the maximum number of iterations is reached. 

**Figure 2:**
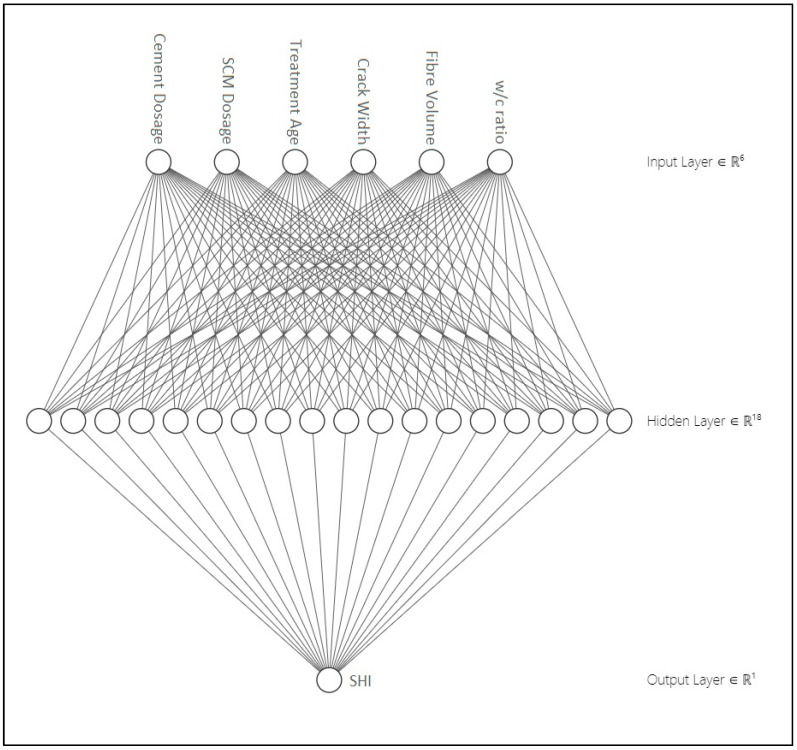
The architecture of the ANN model.

In this ANN model, six input variables have been taken into consideration, i.e., cement dosage (kg/m^3^), SCM dosage (kg/m^3^), fiber content (%vol), *w/c* ratio, crack width (μm), and age of treatment (days). The output variable is SHI. The ANN architecture is presented in Figure 2. During preliminary analysis, firstly, the data corresponding to cases where crack size and age of treatment have not been provided were eliminated. Secondly, the outliers in the data have been detected as elements that are farther than three standard deviations from the mean. MATLAB function “rmoutliers” has been used to remove the outliers. The ANN analysis is carried out using MATLAB “nntool”.

The ANN model has been optimized in terms of several parameters. In this study, the ratio of training to validation to testing subset has been chosen as 0.7:0.15:0.15 [32,37]. The weights are calculated repetitively until they become constant for each cycle. The varied parameters are adaptation learning functions, the number of neurons, and activation function. The optimization algorithm of the backpropagation network used in this study is Levenberg–Marquardt (LM) Algorithm. The least-square non-linear curve fitting problems are solved by the LM algorithm, hence also called a damped least-square method. This algorithm is robust in terms of error coping during the execution of the model. This algorithm is used in MATLAB to solve curve-fitting problems [38]. 

Two adaptation learning functions provided by MATLAB are considered in this study. These two functions are “learngd” and “learngdm”. The “learngd” is the gradient descent (GD) weight and bias learning function while “learngdm” is the gradient descent with momentum (GDM) weight and bias learning function. As the study is preliminary, the number of hidden layers is kept at 1.0 and the number of neurons is varied from 1.0 to 30. The hyperbolic tangent and log-sigmoid functions are used as two activation functions in the neural network model as the only choices provided in MATLAB.

## 3. Results and Discussions

### 3.1. Meta-Data Analysis

Based on the literature review, most of the researchers have investigated the effects of different parameters on the self-healing efficiency and since the data mining analysis is based on the accumulations of the existing study parameters and their effects, the focus has been kept on the effect of the most common studied parameters including crack size, healing time and environment, presence of admixture, and presence of fibers. The meta-data analysis presents the effect of each parameter on the SHI as well as yields confidence on the effect of the parameter on the analyzed SHI.

#### 3.1.1. Crack Size

**Figure 3:**
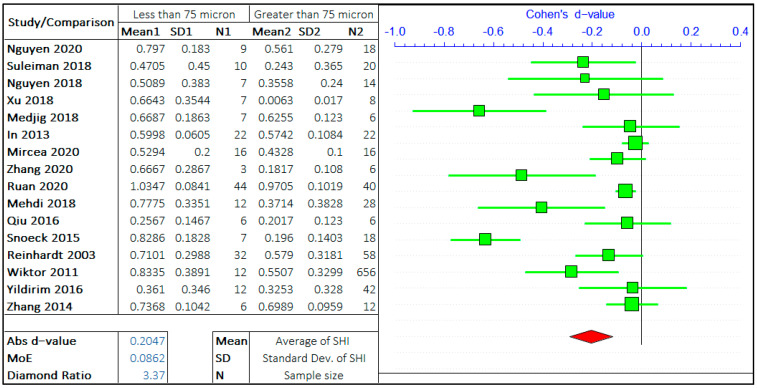
The comparison of self-healing of cracks less than and greater than 75 microns.

**Figure 4:**
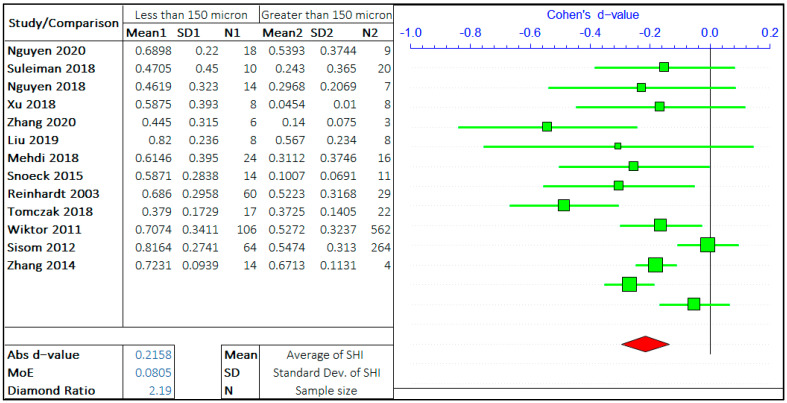
The comparison of self-healing of cracks less than and greater than 150 microns.

**Figure 5:**
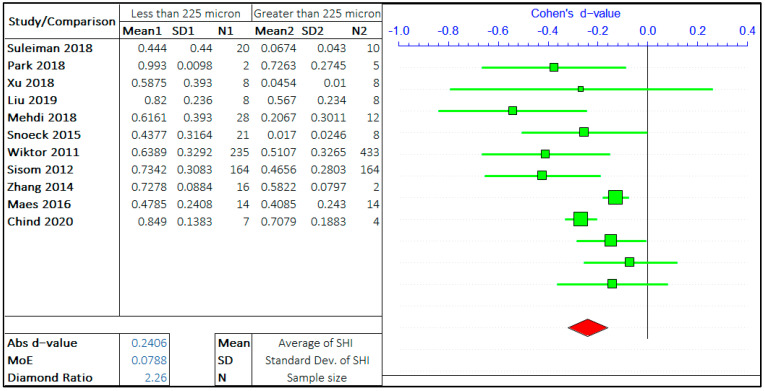
The comparison of self-healing of cracks less than and greater than 225 microns.

A multitude of research has been carried out on the effect of crack size on the self-healing efficiency of concrete. Following the gathering of relevant data and preliminary inspection, the whole range of crack sizes is distributed in a certain range. The argument in this study is the self-healing efficiency of cracks “less than equal to ‘X’ μm” and “greater than ‘X’ μm”. ACI 224R-01 suggests the maximum allowable crack in the range of 100–250 micron for a different type of environment [39]. Hence, the three values of ‘X’ are considered in the given range provided by ACI 224R-01, i.e., 75 μm, 150 μm, and 225 μm. The three ranges assist to analyze the rate of healing with the size of the crack. The meta-analyses of three different arguments are shown in Figure 3, Figure 4 and Figure 5. It should be noted that the negative “*d*-value” on scale presents “less than equal to ‘X’ μm” and “positive *d*-value” represents “greater than ‘X’ μm” case. The total number of relevant studies considered are 16, 13, and 11, for the ‘X’ value of 75 μm, 150 μm, and 225 μm, respectively. It is visually clear from the forest plots that the self-healing efficiency of smaller cracks is better than the bigger cracks. The average “*d*-value” is −0.2203, which shows the faster rate of healing for the smaller crack in all the cases. It is also evident that the absolute value of Cohen’s d is increasing with the increase in the value of ‘X’. The *d*-coefficient, as defined earlier, is proportional to the difference in mean values from two opposing arguments.

Therefore, it is evident that the difference between SHI increases with the increase in crack size with the difference between average SHI for cracks “less than equal to 225 μm” and “greater than 225 μm” being largest. Furthermore, it can be noticed that the difference in *d*-value is also increasing with the crack size. Therefore, this result quantitatively infers that the SHI value decreases at a faster rate with the increase in crack size.

The MoE values are of the same order for all three studies showing the equal similar variance in data for three analyses. The average value of the diamond ratio is calculated as 2.61, which shows the presence of heterogeneity in data collected from different studies. This can be attributed to the variation of other parameters, that aspect of the self-healing efficiency of concrete, among multiple studies.

#### 3.1.2. Healing Period

**Figure 6:**
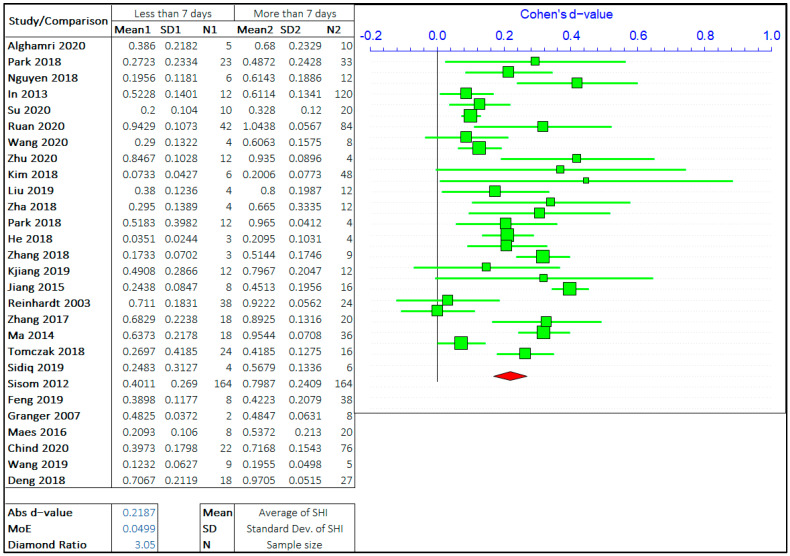
The comparison of self-healing of cracks less than and greater than 7 days.

**Figure 7:**
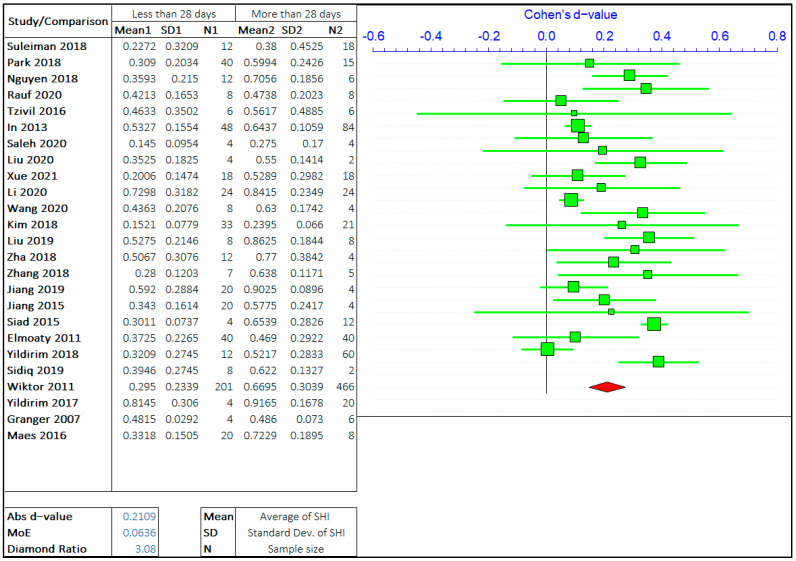
The comparison of self-healing of cracks less than and greater than 28 days.

**Figure 8:**
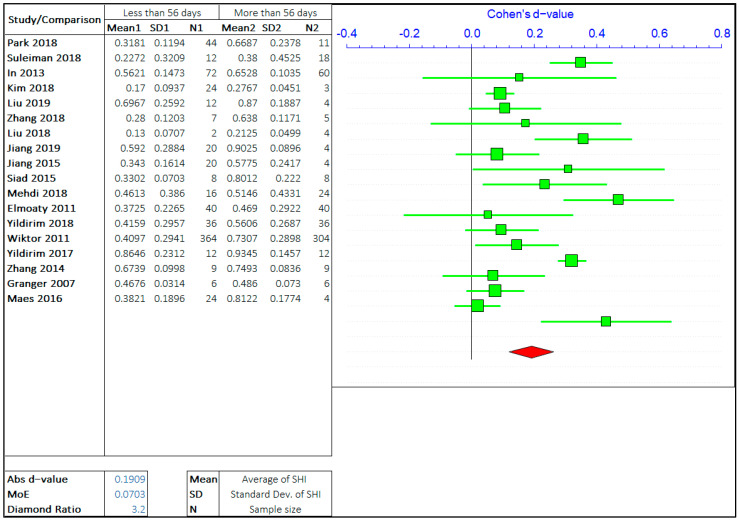
The comparison of self-healing of cracks less than and greater than 56 days.

The effect of the healing period on self-healing is the most researched parameter on the self-healing efficiency of cement-based materials. The self-healing time has been varied from few hours to 1 year and even more depending on the employed healing technology. However, most studies focus on the healing period of 3 days to 90 days. Imitating the same steps as in the case of cracks, the overall range of healing time has been divided by three values based on the observations from multiple studies. The opposing arguments will be the self-healing capacity of concrete treated for “less than or equal to ‘X’ days” and “greater than ‘X’ days”. The three values of ‘X’ chosen are 7 days, 28 days, and 56 days. The meta-analyses of three different arguments are shown in Figure 6, Figure 7 and Figure 8. It should be noted that the “negative *d*-value” on the scale presents “less than equal to ‘X’ days” and “positive *d*-value” represents “greater than ‘X’ days” case. The total number of relevant studies considered are 28, 25, and 18, for ‘X’ value of 7 days, 28 days, and 56 days, respectively.

It can be observed in the forest plots that the self-healing efficiency for a longer healing time is better than for shorter periods. The average “*d*-value” is 0.2068, which shows the faster rate of healing for a longer amount of treatment time. It is also evident that the *d*-value is decreasing with the increase in the value of ‘X’. It is inferred that the difference between SHI decreases with the increase in the healing period with the difference between average SHI for the period of “less than equal to 7 days” and “greater than 7 days” being largest. Furthermore, it can be noticed that the difference in *d*-value is also increasing with the healing period. Therefore, this result quantitatively suggests that the rate of healing decreases with an increase in the healing period. The healing rate is higher in the early stage, as autogenous healing happens predominantly in the first 28 days.

The average value of the diamond ratio is calculated as 3.11, which shows the presence of heterogeneity in data collected from different studies. The reasons have been explained in the previous section. It is also evident from the average “*d*-value” that the significance of the effect of crack size is like the effect of the healing period on the self-healing capacity of concrete.

#### 3.1.3. Crystalline Admixtures

**Figure 9:**
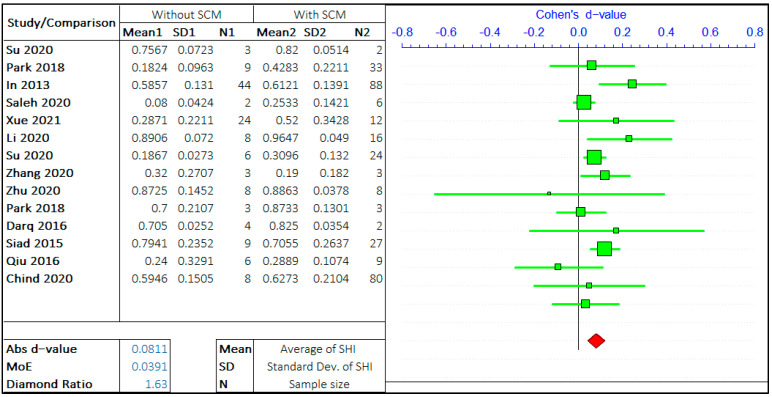
The effect of SCM on the self-healing capacity of concrete.

**Figure 10:**
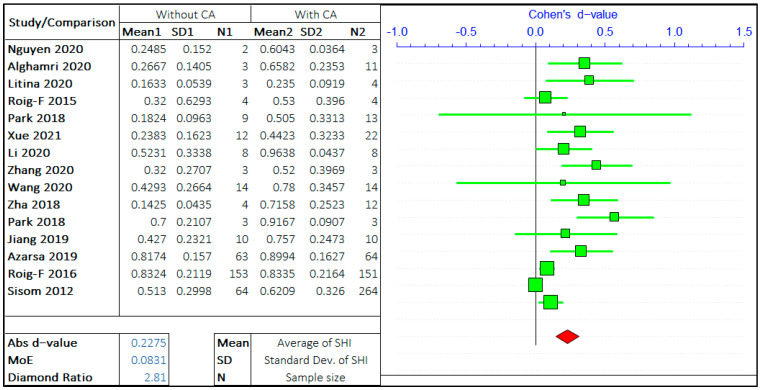
The effect of crystalline admixture (CA) on the self-healing capacity of concrete.

In this study, the effect of SCM and the effect of crystalline admixtures (CA) have been studied separately. In both the studies, the opposing arguments are the self-healing capacity of concrete “without admixture” and “with admixture”. The meta-analysis of two different admixtures are shown in Figure 9 and Figure 10. It should be noted that the negative “*d*-value” on scale represents “without admixture” and positive “*d*-value” represents the “with admixture” case. The total number of relevant studies considered are 14 and 15, for mineral and chemical admixture, respectively. 

The forest plots show that the self-healing efficiency of concrete containing the admixtures is higher than for concrete without the presence of admixtures. The *d*-value for analysis with SCM and CA are 0.0811 and 0.2275, respectively. This striking difference between these two values shows the upper hand of crystalline admixtures on improving the self-healing capacity of concrete. The presence of CA in the concrete mix design is a significant factor equivalent to, if not more than, the crack size and healing period. A very low *d*-value infers the improvement of SHI with the inclusion of SCM; however, its effect is not as significant compared to the presence of CA. The average value of the diamond ratio is calculated as 2.22, which shows the presence of heterogeneity in data collected from different studies. The reasons have been explained in the previous sections.

#### 3.1.4. Healing Environment

The healing environment significantly influences the self-healing efficiency of concrete. The studies have been carried out with different treatment conditions, such as complete water immersion, wet/dry cycles, sea-water exposure, humid chamber, air curing, natural weathering, etc. However, two of the most common methods are water immersion and wet/dry cycles. The analyses of all the treatment conditions have not been possible due to the lack of multiple studies. Therefore, the meta-data analysis has been carried out to compare the effect of water curing with the effect of the wet/dry cycle on the self-healing capacity of concrete.

**Figure 11:**
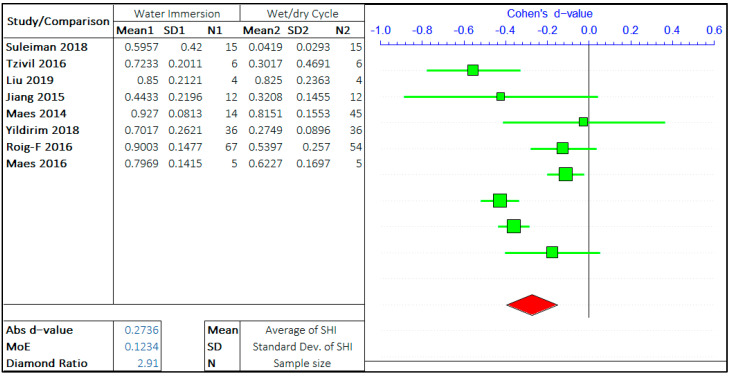
The effect of treatment environment on the self-healing capacity of concrete.

The two arguments in this study are “Water immersion” and “Wet/Dry cycle”. The meta-analysis has been presented in Figure 11. The positive *d*-value supports the wet/dry cycles, and the negative value supports the complete water immersion. The total number of studies considered is limited to 8. The forest plots show that the self-healing efficiency of concrete incomplete water immersion is far greater than the wet/dry cycles. The *d*-value for analysis is −0.2736, quite relevant if compared to all the other values obtained in the analysis. This shows the high significance of the healing environment on the self-healing capacity of the cementitious system, more importantly, the time of exposure to water.

The MoE value is 0.1234, which shows a high variance in the errors, mainly due to a smaller number of relevant studies on the same topics. The average value of the diamond ratio is calculated as 2.91, which shows the presence of heterogeneity in data collected from different studies. The reasons have been explained in the previous sections.

#### 3.1.5. Presence of Fibres

The effect of the presence of fibers on autogenous self-healing of concrete has been explored by several researchers. The type of fibers has been varied, including PVA fibers, PP fibers, steel fibers of different shapes, acrylic fibers, etc. However, there are not a significant number of studies with the variation of fiber content and type. Therefore, all the types of fiber have been grouped in a single category of fiber. The meta-analysis has been carried out to study the effect of the presence of any fiber on the SHI of concrete.

**Figure 12:**
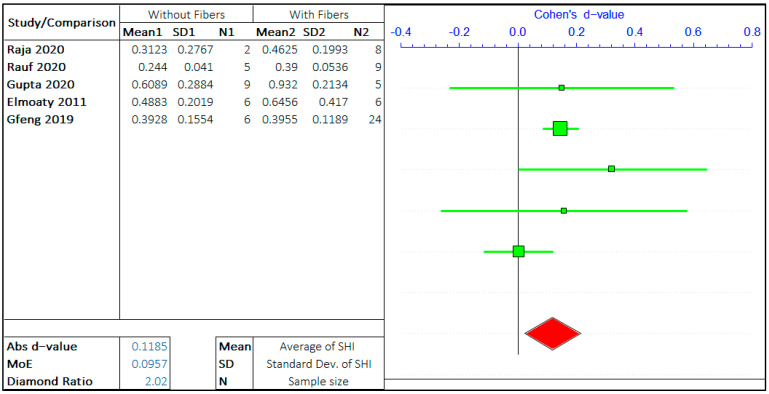
The effect of fiber’s presence on the self-healing capacity of concrete.

The two arguments in this study are “Without fiber” and “With fiber”. The meta-analysis is presented in Figure 12. The positive *d*-value supports the presence of fiber and the negative value supports the absence of fibers. The total number of studies considered is limited to 5. 

The forest plots show that the self-healing efficiency of concrete with the presence of fiber is slightly better than the concrete without fiber. The *d*-value for analysis is 0.1185. This is a small value compared to all the other values obtained in the analysis, highlighting the low significance of fiber on the self-healing capacity of the cementitious system. The MoE value is 0.0957, which shows a high variance in the errors. This is mainly because of a smaller number of relevant studies on the same topics. The average value of the diamond ratio is calculated as 2.02, which shows the presence of heterogeneity in data collected from different studies. The reasons have been explained in the previous sections.

#### 3.1.6. Meta-Data Analysis: Comparisons

Forest plots take consideration of numerous studies to show the effect of each parameter on the SHI at a general scale. Cohen’s *d*-value shows the effect of parameter on design and MoE yields the confidence on the effect of parameter on SHI. Table 2 presents the comparison of the impact of the parameter and the confidence of the impact of each parameter. It is relevant that the treatment condition, crystalline admixture, and crack size have the highest influence on the SHI. However, fiber content and SCM has the lowest impact on the SHI. The confidence ranking establishes that period of healing and cracks size are the most reliable parameters to have an impact on the SHI of cementitious material. 

It is noted that the meta-data analysis portrays that the SCM content does not impact the SHI significantly; however, the meta-analysis only considers the presence or the absence of SCM in the mix design. In most of the studies, cement has been replaced by a certain percentage of SCM. Hence, the conclusion of meta-analysis is not dependable as the amount of cement is variable during the comparison of data. Therefore, a relative importance analysis has been carried out keeping the total cementitious material (TCM) as a parameter to provide a better picture.

### 3.2. Relative Importance 

**Figure 13:**
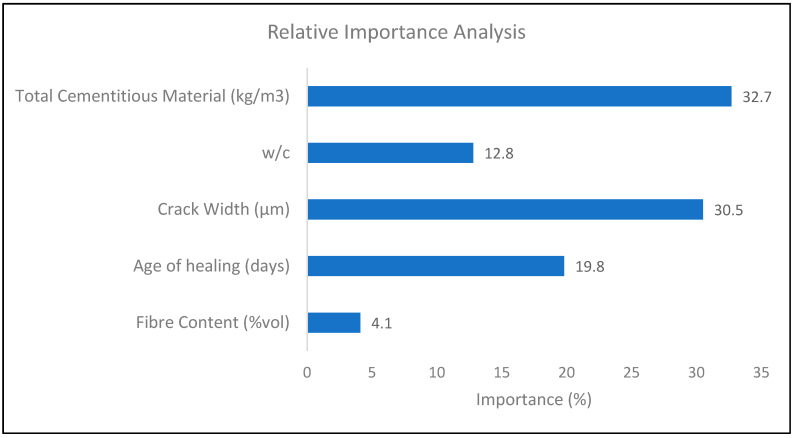
Relative importance analysis.

The relative weight analysis is an effective method to evaluate the relative importance of correlated parameters. Figure 13 represents the results obtained from the analysis. It is observed that total cementitious material in concrete has the greatest influence on the improvement of SHI, with an importance percentage of 32.7%. The crack width has a similar influence on SHI as the amount of cementitious material with an importance factor of 30.5%. It has also been shown through the meta-analysis that the crack width has a high influence on the self-healing capacity of the concrete. Hence, this analysis corroborates the result of the meta-analysis. 

The age of healing and water to cement content has also a significant effect on determining the SHI with an importance percentage of around 20% and 13%, respectively. The results validate the statements made based on the meta-analysis regarding the healing period in the previous sections of the study. Similarly, the relative importance percentage of fiber content is lowest with a percentage of 4.1%, confirming the low *d*-value result from the meta-analysis hence the low influence of the fiber content on the self-healing capacity of concrete.

### 3.3. Optimization Charts

**Figure 14:**
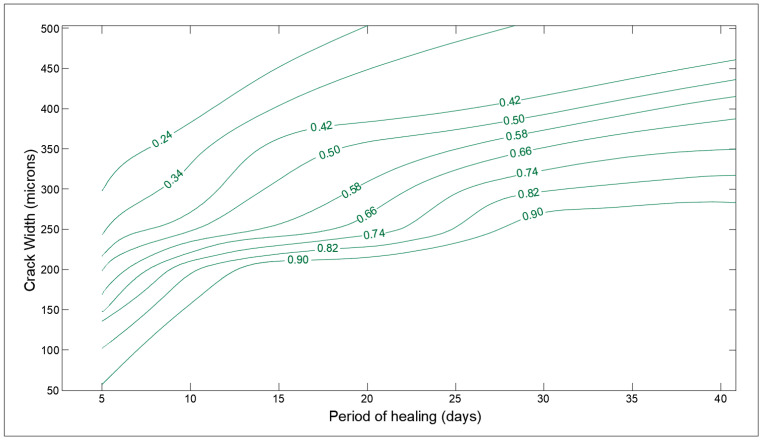
Optimization chart for crack width (microns) and period of healing (days).

**Figure 15:**
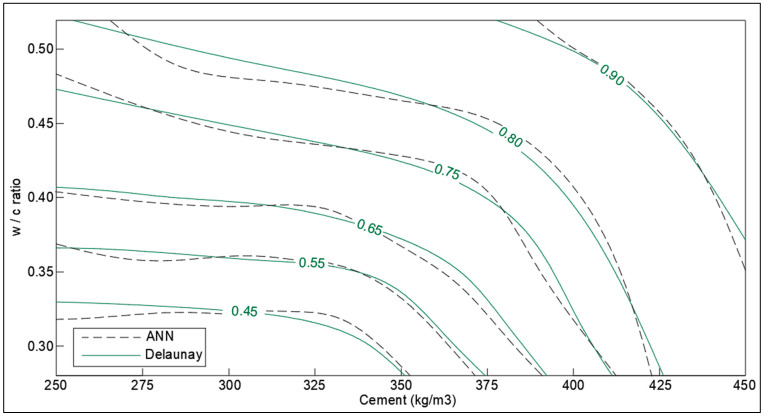
Optimization chart for *w/c* ratio and cement (kg/m^3^) (with ANN estimation).

**Figure 16:**
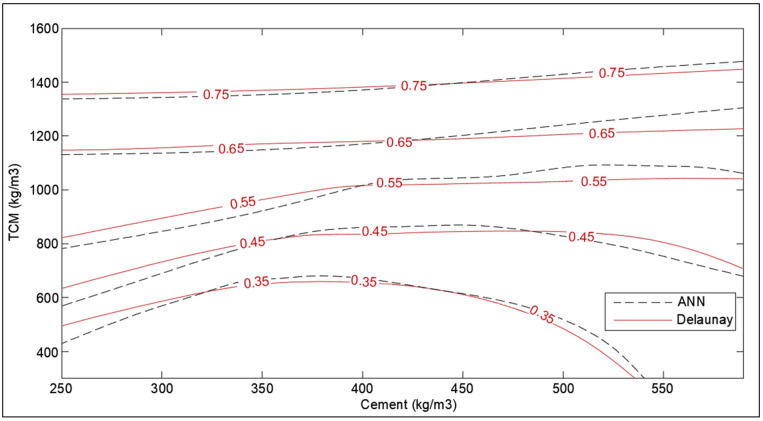
Optimization chart for TCM (kg/m^3^) and cement (kg/m^3^) (with ANN estimation).

**Figure 17:**
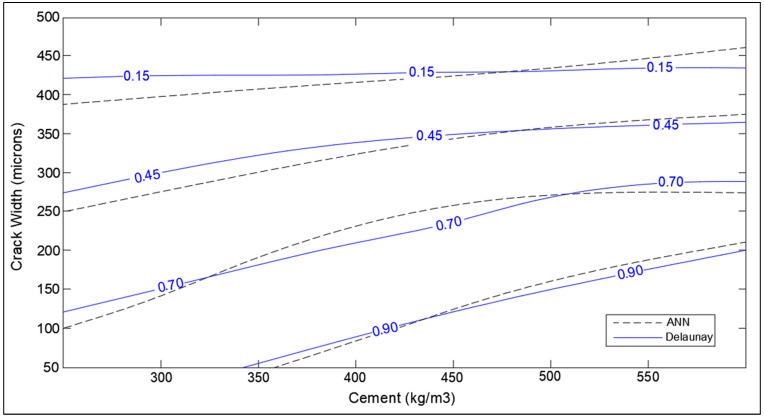
Optimization chart for crack width (micron) and cement (kg/m^3^) (with ANN estimation).

**Figure 18:**
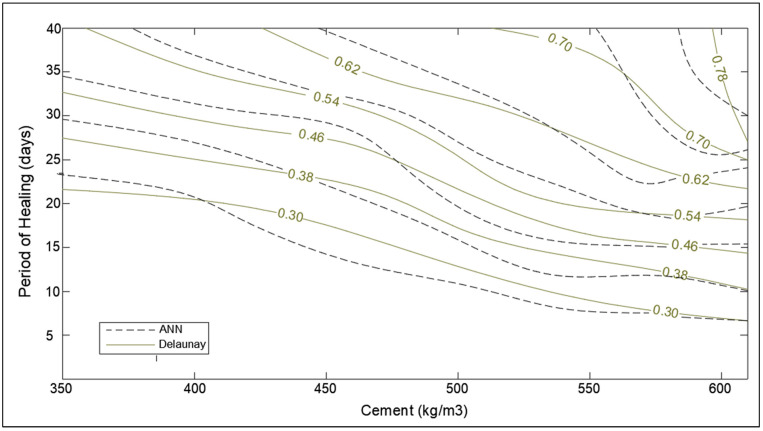
Optimization chart for the period of healing (days) and cement (kg/m^3^) (with ANN estimation).

**Figure 19:**
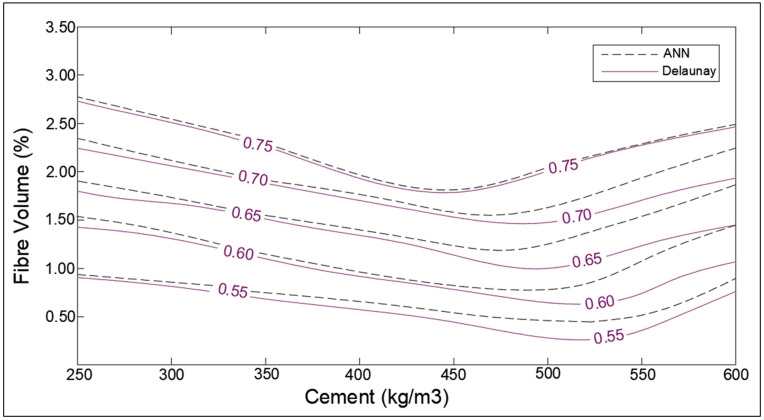
Optimization chart for fibre volume (%) and cement (kg/m^3^) (with ANN estimation).

The optimization charts of some of the design parameters are shown in Figure 14, Figure 15, Figure 16, Figure 17, Figure 18 and Figure 19. The influence of crack width and period of healing (immersed in water) on the SHI is finally shown in Figure 14. The cracks smaller than 100 microns heal almost completely, even after a short period of treatment as anticipated by several studies [40]. Furthermore, it is observed that the rate of healing decreases notably after a period of 14 to 28 days of treatment depending on the crack size. This can be attributed to the fact that the degree of hydration in concrete rapidly increases until 28 days and then becomes constant [41]. Furthermore, this behavior is supported by the claims made by meta-analysis based on numerous studies in Section 3.1.

The SHI contours for the *w/c* ratio and the dosage of cement (kg/m^3^) in concrete are presented in Figure 15. The results obtained depend on the composition of concrete, generally having a high *w/c* ratio for low-cement concrete (low-quality concrete) and vice versa (high-performance concrete). The SHI also gets significantly influenced by other factors like the period of healing and width of cracks. It is noticeable from the chart that SHI increases with cement content even with low *w/c* ratios, which is reasonable because this will correspond to the higher availability of un-hydrated cement for further hydration reactions. However, the rate of increase in SHI decreases slightly with the increase in cement content in concrete.

The effect of supplementary cementitious material on the SHI is presented in Figure 16 using cement content and the amount of total cementitious material. As anticipated, the SHI increases with the amount of total cementitious material in concrete. However, it can be noted that there is a peak range for SCM content for the given amount of total cementitious material for optimum SHI. Following the peak, the increase in cement content in total cementitious constituent is preferred for improved SHI. This can be attributed to the thermo-chemistry of hydration reaction of the cement–fly ash blend in concrete. The hydration reaction of fly-ash utilizes the hydration products of cement, namely the calcium hydroxide to produce new calcium silicate hydrates [42]. Hence, the low amount of cement in the blend results in a lack of activation of the hydration reaction of fly-ash. This can be overcome with the addition of activators like sodium hydroxide and sodium silicate in the mix-design [43].

The influence of crack width on self-healing efficiency is presented in Figure 17. It is evident from the chart that small cracks (50–100 micron) have high SHI, around 0.75 to 1, even in concrete with low cement content. The SHI index drops significantly with higher crack size, going as low as 0.15 for concrete with cracks of 400 microns. The effect of cement content is distinct in the smaller cracks, where cracks heal with a higher healing index in high-cement concrete. However, this trend appears to disappear for cracks higher than 350 microns. This behavior implies that the additional un-hydrated cement does not facilitate the healing of large cracks, which is not corroborated or implied by any existing literature. This behavior may be attributed to interpolation from limited data points due to the lack of studies showing the healing performance of larger cracks in high-cement concrete.

The contour graph of SHI mapping the effect of the period of healing as well as cement content is presented in Figure 18. As anticipated from multiple works of literature, self-healing improves with both periods of healing and cement content in concrete [6,7]. The rapid increase in SHI with the period of healing also owes to the underlying selection of data corresponding to completely submerged environmental conditions. For a longer period of healing, the effect of cement becomes more pronounced on healing capacity as the hydration mechanism is almost complete following the initial 28 days of the interaction of cement and water [41]. Therefore, for a longer period of exposure, the SHI depends on the time water takes to reach the available un-hydrated cement in numerous pores. Hence, high-cement concrete exposed to a long period of healing yields a high degree of hydration, and so high self-healing indices. Moreover, the SHI becomes practically independent of the period of exposure.

The influence of fiber content with cement on the self-healing capacity of concrete is shown in Figure 19. The increase in the amount of fiber causes a slight improvement in the SHI of concrete. It is visible from the charts that for low-cement concrete, the increase in SHI due to the addition of fiber is slightly more pronounced than high-cement concrete. The fibers control the opening of the cracks that make self-healing products easily attach to the surface of the crack and fill them. A peculiar behavior has been shown by the chart in which the healing indices seem to decrease with cement for high-cement concrete for a particular volume of fiber. This can be due to the influence of other significant parameters like large crack size or low treatment period.

It has any way to be remarked that, since the optimization charts are based on the available reliable literature, they only cover a specific range of parameters. Therefore, more range of parameters should be included and an effort from the scientific community being implemented in documenting in the papers, as many of them as possible to enable meta-analysis to cover a wider range of applicability, thus also paving the way for the construction of a vigorous database that could foster standardization of healing test methods and design-oriented healing efficacy assessment procedures. Furthermore, besides estimating SHI using the Delaunay triangulation, a multi-input and output neural network model is being developed and optimized using the collected data. The development of the model and inferences are discussed in the following section.

### 3.4. Artificial Neural Network 

**Figure 20:**
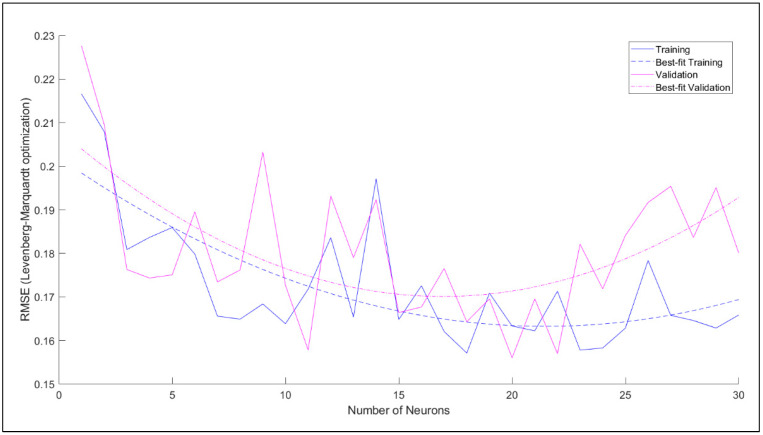
RMSE value against the number of neurons for LM training algorithm.

The step-wise optimization of the ANN model has been performed in the following steps. Firstly, the Root Means Square Error (RMSE) values were calculated for the optimization of the number of neurons. Figure 20 shows the RMSE value against the number of neurons for the LM training algorithm. It is evident from Figure 20 that the lowest RMSE of the training set and validation set such that RMSE of validation set is slightly higher than the training set is for 18 neurons in the hidden layer. Therefore, the 18 neurons in the hidden layer were adopted for further investigation.

**Figure 21:**
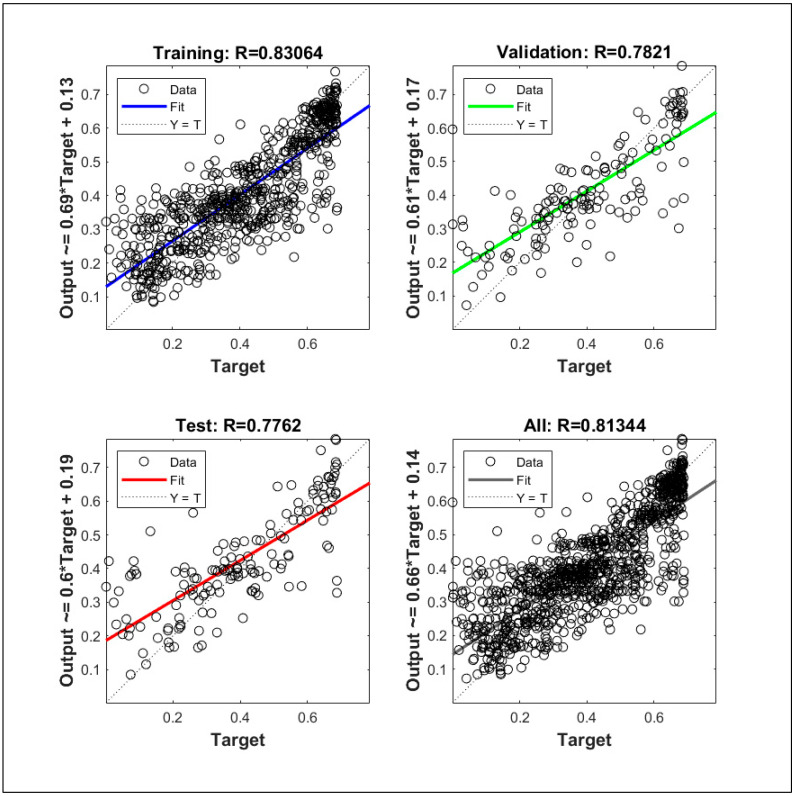
Regression model for gradient descent (momentum) and hyperbolic tangent sigmoid.

The optimization corresponding to adaptation learning functions and activation functions were also performed concerning correlation coefficient, R. The two adaptation learning functions were gradient descent (learngd) and gradient descent with momentum (learngdm), while two activation functions were hyperbolic tangent sigmoid (tansig) and hyperbolic tangent (tanh) function. The four combinations based on two parameters were hence learngd-tansig, learngd-tanh, learngdm-tansig, and learngdm-tanh. The best regression model was obtained for the learngdm-tansig combination (Figure 21). The design charts were generated using the optimized model and compared with the charts obtained from Delaunay’s approximation (Figure 15, Figure 16, Figure 17, Figure 18 and Figure 19). This further investigation yields a robust mechanism that would provide a more accurate approximation of SHI based on all the input parameters involved. The following passage explains more about design charts from the ANN model.

The SHI contour plot based on crack size and cement content (Figure 17) is in good agreement with the theoretical interpolation chart. It is interesting to note that the neural network model is showing the dependence of healing capacity on the amount of cement in high-cement concrete contrary to interpolation approximation. This is because neural networks consist of several nodes that provide a better estimate than Delaunay triangulation interpolation. In the case of total cementitious material and *w/c* ratio in Figure 15 and Figure 16, the model provides a very good correlation with the interpolation data with the small error at the extreme ends.

In the case of healing period and fiber volume, the performance trends from the chart obtained (Figure 18 and Figure 19) from ANN are similar to trends obtained from Delaunay approximation; however, it is evident that error is moderately higher than the three latter cases. The reason for the error in the estimation of SHI based on fiber content can be associated with the relative lack of data points for concrete samples containing fibers. Furthermore, the study lacked the demarcation of fiber type [44,45], and its orientation in concrete due to a lack of substantial literature. The different types of fibers have a varying effect on the concrete, hence grouping all together may cause inconsistencies in estimation. To overcome this problem, more data regarding the fiber type, size, and orientation must be collected and trained to develop a more powerful model. The lack of parameters considered may be the reason for the observed error in the chart providing SHI based on the healing period. These parameters involve the temperature of water [7], solute in water (like NaCl, sulfates, chlorides, etc.) [46], and gaseous content [47]. For a more comprehensive study, demarcated data is required to minimize the obtained error. The ANN model has been developed with the objective of developing a robust input–output model to calculate SHI based on the considered parameters. The model provides results closer to theoretical values (as shown by the charts); however, there is significant error due to low correlation value (*R*-value) of the model. Hence, it is necessary to improve the ANN model by improving it as a multi-layer deep neural network model to provide better approximation of SHI value.

## 4. Conclusions

A systematic review of 100 papers has been carried out to study the effect of several factors on the self-healing capacity of cementitious systems. Primarily, a preliminary literature review has been carried out to investigate the factors that influence the autogenous self-healing efficiency of concrete. The numerical values of these factors and corresponding self-healing indices (calculated) are obtained to carry on quantitative analysis. The summary of important finding corresponding to meta-analysis, design charts, and neural network model is presented below:

1. The statistical analysis has been carried out using statistical tools, like effect size, the margin of error, and the diamond ratio. The meta-analysis shows that crack size, healing period and environment, as well as the presence of CA has the highest effect on the self-healing efficiency. On the other hand, presence of fibers and SCM improves the self-healing capacity in cementitious materials; however, the effect is not as significant as other parameters.
2. Based on the optimization charts, this study can suggest some recommendations and highlight the expected research works that would contribute to fill the gap and provide more data to strengthen the confidence relating to self-healing/sealing parameters. A brief analysis is drawn below.
  - The relative importance percentage of total cementitious material, crack width, period of healing, *w/c* ratio, and fiber content in determining the SHI are 32.7%, 30.5%, 20%,13%, and 4.1%, respectively. The results validate the conclusions made based on the meta-analysis.
  - The analysis suggests that the crack greater than 300 microns have much less healing efficiency, even for longer times of treatment, whereas cracks smaller than 100 microns can heal completely within a shorter rime, down to one week. Furthermore, it is observed that the rate of healing decreases notably after a period of 14 to 28 days of treatment depending on the crack size, hence corroborating with the reaction mechanism of autogenous self-healing in concrete.
  - The design chart of *w/c* ratio and cement content shows that SHI increases with cement content even with low *w/c* ratios. This is because the hydration reactions augment with the availability of un-hydrated cement. However, more data is required for low-cement concrete with high *w/c* ratio to obtain better trends in optimization charts.
  - The analysis of supplementary cementitious material shows that there is a peak range for SCM content, around 40–60% of total cementitious materials, for optimum SHI. This is because the hydration reaction of fly-ash utilizes the hydration products of cement. The low amount of cement produces less hydration energy for the activation of the hydration reaction of fly-ash.
  - The design chart investigating the effect of the period of healing shows rapid recovery at the beginning of the treatment process. For a longer period of healing, the effect of cement becomes more pronounced on healing capacity. It is suggested to treat concrete for the period of around 28 days as the hydration mechanism is almost complete in this period.
  - The increase in the volume of fibers from 1% to 2.5% causes a slight improvement in the SHI of concrete. It has been shown that for low-cement concrete, the increase in SHI due to the addition of fibers is slightly more pronounced than high-cement concrete.
  - The results obtained were in good agreement with the existing literature. However, more range of parameters should be included, and more relevant papers should be documented to enable meta-analysis to cover a wider range of applicability.
3. The results of neural network modeling are presented as follows.
  - The SHI contour plots based on crack size, total cementitious material, *w/c* ratio are in good agreement with the theoretical interpolation charts, whereas a moderate error has been observed in the charts of the healing period and fiber volume. This can be attributed to the lack of demarcation of data points based on more parameters.
  - It is evident that optimized single-layer feed forward neural network provides a low correlation value. Hence, multilayered deep model must be developed in future study to improve the accuracy of the machine learning model.

The approach herein employed, together with the produced design charts can pave the way toward codification and standardization of the composition of cementitious materials to exploit its best self-healing capacity. 

## Figures and Tables

**Table 1:** Self-healing indices formulas for different experiments.

S. No.	Experiments	Parameters	Formulas
1	Crack Size	$w_{i}$ = initial width & $w_{f}$ = final width	$(w_{i}-w_{f})/w_{i}$
2	Permeability Test	$Q_{i}$ = initial flow & $Q_{f}$ = final flow	$(Q_{i}-Q_{f})/Q_{i}$
3	Ultrasonic Pulse Velocity (UPV) Test	$U_{h}$ = UPV of healed sample & $U_{c}$ = UPV of cracked sample	$(U_{h}-U_{c})/U_{h}$
4	Mechanical Strength	$M_{h}$ = Strength of healed sample & $M_{c}$ = Strength of cracked sample	$(M_{h}-M_{c})/M_{h}$

**Table 2:** Comparison of the impact of parameters and the corresponding confidence on their impact.

Parameter	Avg. *d*-Value (Absolute)	Impact Ranking	Average MoE	Confidence Ranking
Crack Size	0.22	3	0.082	2
Period of Healing	0.207	4	0.061	1
SCM	0.081	6	0.039	5
CA	0.227	2	0.083	3
Treatment Condition	0.274	1	0.123	4
Fiber Content	0.118	5	0.096	6

## Data Availability

The supplementary material contained the complete list of all the sources from which the concrete self-healing data were compiled.

## Supplementary Materials

The following are available online at https://www.mdpi.com/article/10.3390/ma14164437/s1. Supplementary material—Database and Validation Set: List of Sources.
