# Supplementary material for: Meta-Analysis and Machine Learning Models to Optimize the Efficiency of Self-Healing Capacity of Cementitious Material
Source: Materials (Basel). 2021 Aug 8;14(16):4437. doi: 10.3390/ma14164437 (PMC8398163; doi:10.3390/ma14164437)
Supplement: Supplementary file 1 [file materials-14-04437-s001.zip › materials-1301230-supplementary.pdf]

materials

MDPI

Supplemental File

Meta-Analysis and Machine Learning Models to Optimize the Efficiency of Self-Healing Capacity of Cementitious Material

Shashank Gupta [1,*], Salam Al-Obaidi [1,2] and Liberato Ferrara [1]

DATABASE AND VALIDATION DATASET –LIST OF SOURCES

The following 100 articles are used to carry out the meta-data and ANN analysis.

1. Abd, E.M. Self-Healing of Polymer Modified Concrete. 8.

2. Alghamri, R.; Al-Tabbaa, A. Self-Healing of Cracks in Mortars Using Novel PVA-Coated Pellets of Different Expansive Agents. Construction and Building Materials 2020, 254, 119254.

3. Araújo, M.; Van Tittelboom, K.; Dubruel, P.; Van Vlierberghe, S.; De Belie, N. Acrylate-Endcapped Polymer Precursors: Effect of Chemical Composition on the Healing Efficiency of Active Concrete Cracks. Smart Materials and Structures 2017, 26, 055031.

4. Azarsa, P.; Gupta, R.; Biparva, A. Assessment of Self-Healing and Durability Parameters of Concretes Incorporating Crystalline Admixtures and Portland Limestone Cement. Cement and Concrete Composites 2019, 99, 17–31.

5. Chindasiriphan, P.; Yokota, H.; Pimpakan, P. Effect of Fly Ash and Superabsorbent Polymer on Concrete Self-Healing Ability. Construction and Building Materials 2020, 233, 116975.

6. Darquennes, A.; Olivier, K.; Benboudjema, F.; Gagné, R. Self-Healing at Early-Age, a Way to Improve the Chloride Resistance of Blast-Furnace Slag Cementitious Materials. Construction and Building Materials 2016, 113, 1017–1028.

7. Deng, H.; Liao, G. Assessment of Influence of Self-Healing Behavior on Water Permeability and Mechanical Performance of ECC Incorporating Superabsorbent Polymer (SAP) Particles. Construction and Building Materials 2018, 170, 455–465.

8. Du, W.; Yu, J.; Gu, Y.; Li, Y.; Han, X.; Liu, Q. Preparation and Application of Microcapsules Containing Toluene-Di-Isocyanate for Self-Healing of Concrete. Construction and Building Materials 2019, 202, 762–769.

9. Feng, J.; Su, Y.; Qian, C. Coupled Effect of PP Fiber, PVA Fiber and Bacteria on Self-Healing Efficiency of Early-Age Cracks in Concrete. Construction and Building Materials 2019, 228, 116810.

10. Gao, S.; Jin, J.; Hu, G.; Qi, L. Experimental Investigation of the Interface Bond Properties between SHCC and Concrete under Sulfate Attack. Construction and Building Materials 2019, 217, 651–663.

11. Granger, S.; Loukili, A.; Pijaudier-Cabot, G.; Chanvillard, G. Experimental Characterization of the Self-Healing of Cracks in an Ultra High Performance Cementitious Material: Mechanical Tests and Acoustic Emission Analysis. Cement and Concrete Research 2007, 37, 519–527.

12. Gray, R.J. Autogenous Healing of Fibre/Matrix Interfacial Bond in Fibre-Reinforced Mortar. Cement and Concrete Research 1984, 14, 315–317.

13. Gupta, S.; Dai Pang, S.; Kua, H.W. Autonomous Healing in Concrete by Bio-Based Healing Agents–A Review. Construction and Building Materials 2017, 146, 419–428.

14. Guzlena, S.; Sakale, G. Self-Healing of Glass Fibre Reinforced Concrete (GRC) and Polymer Glass Fibre Reinforced Concrete (PGRC) Using Crystalline Admixtures. Construction and Building Materials 2021, 267, 120963.

15. He, H.; Zhu, Y.; Zhou, A. Electrochemical Impedance Spectroscopy (EIS) Used to Evaluate Influence of Different External Pressures, Curing Ages and Self-Healing Environments on the Self-Healing Behavior of Engineered Cementitious Composites (ECC). Construction and Building Materials 2018, 188, 153–160.

16. Homma, D.; Mihashi, H.; Nishiwaki, T. Self-Healing Capability of Fibre Reinforced Cementitious Composites. Journal of Advanced Concrete Technology 2009, 7, 217–228.

17. Hong, G.; Choi, S. Rapid Self-Sealing of Cracks in Cementitious Materials Incorporating Superabsorbent Polymers. Construction and Building Materials 2017, 143, 366–375.

18. Hung, C.-C.; Su, Y.-F. Medium-Term Self-Healing Evaluation of Engineered Cementitious Composites with Varying Amounts of Fly Ash and Exposure Durations. Construction and Building Materials 2016, 118, 194–203.

19. Hung, C.-C.; Su, Y.-F.; Hung, H.-H. Impact of Natural Weathering on Medium-Term Self-Healing Performance of Fiber Reinforced Cementitious Composites with Intrinsic Crack-Width Control Capability. Cement and Concrete Composites 2017, 80, 200–209.

20. In, C.-W.; Holland, R.B.; Kim, J.-Y.; Kurtis, K.E.; Kahn, L.F.; Jacobs, L.J. Monitoring and Evaluation of Self-Healing in Concrete Using Diffuse Ultrasound. NDT & E International 2013, 57, 36–44.

21. Jiang, Z.; Li, W.; Yuan, Z. Influence of Mineral Additives and Environmental Conditions on the Self-Healing Capabilities of Cementitious Materials. Cement and Concrete Composites 2015, 57, 116–127.

22. Jiang, Z.; Yuan, Z.; Li, W. Acoustic Emission Analysis of Characteristics of Healing Products in Steam-Cured Cementitious Materials with Mineral Additives. Construction and Building Materials 2019, 201, 807–817.

23. Kan, L.; Shi, H. Investigation of Self-Healing Behavior of Engineered Cementitious Composites (ECC) Materials. Construction and Building Materials 2012, 29, 348–356.

24. Khaliq, W.; Ehsan, M.B. Crack Healing in Concrete Using Various Bio Influenced Self-Healing Techniques. Construction and Building Materials 2016, 102, 349–357.

25. Kim, H.G.; Qudoos, A.; Ryou, J.-S. Self-Healing Performance of GGBFS Based Cementitious Mortar with Granulated Activators Exposed to a Seawater Environment. Construction and Building Materials 2018, 188, 569–582.

26. Kim, S.; Yoo, D.-Y.; Kim, M.-J.; Banthia, N. Self-Healing Capability of Ultra-High-Performance Fiber-Reinforced Concrete after Exposure to Cryogenic Temperature. Cement and Concrete Composites 2019, 104, 103335.

27. Kobayashi, K.; Suzuki, M.; Rokugo, K. The Effects of PE and PVA Fiber and Water Cement Ratio on Chloride Penetration and Rebar Corrosion Protection Performance of Cracked SHCC. Construction and Building Materials 2018, 178, 372–383.

28. Kwon, S.; Nishiwaki, T.; Kikuta, T.; Mihashi, H. Experimental Study on Self-Healing Capability of Cracked Ultra-High-Performance Hybrid-Fiber-Reinforced Cementitious Composites. In Proceedings of the 3rd International Conference on Sustainable Construction Materials and Technologies, Kyoto, Japan; 2013.

29. Lee, H.X.D.; Wong, H.S.; Buenfeld, N.R. Self-Sealing of Cracks in Concrete Using Superabsorbent Polymers. Cement and Concrete Research 2016, 15.

30. Lee, H.X.D.; Wong, H.S.; Buenfeld, N.R. Potential of Superabsorbent Polymer for Self-Sealing Cracks in Concrete. 8.

31. Li, G.; Liu, S.; Niu, M.; Liu, Q.; Yang, X.; Deng, M. Effect of Granulated Blast Furnace Slag on the Self-Healing Capability of Mortar Incorporating Crystalline Admixture. Construction and Building Materials 2020, 239, 117818.

32. Litina, C.; Al-Tabbaa, A. First Generation Microcapsule-Based Self-Healing Cementitious Construction Repair Materials. Construction and Building Materials 2020, 255, 119389.

33. Liu, H. Effects of External Multi-Ions and Wet-Dry Cycles in a Marine Environment on Autogenous Self-Healing of Cracks in Cement Paste. Cement and Concrete Research 2019, 9.

34. Liu, H. Promotion on Self-Healing of Cracked Cement Paste by Triethanolamine in a Marine Environment. Construction and Building Materials 2020, 10.

35. Liu, H. Self-Healing of Microcracks in Engineered Cementitious Composites under Sulfate and Chloride Environment. Construction and Building Materials 2017, 9.

36. Ma, H. Effect of Self-Healing on Water Permeability and Mechanical Property of Medium-Early-Strength Engineered Cementitious Composites. Construction and Building Materials 2014, 10.

37. Maes, M. Chloride Penetration in Cracked Mortar and the Influence of Autogenous Crack Healing. Construction and Building Materials 2016, 11.

38. Medjigbodo, S.; Bendimerad, A.Z.; Rozière, E.; Loukili, A. How Do Recycled Concrete Aggregates Modify the Shrinkage and Self-Healing Properties? Cement and Concrete Composites 2018, 86, 72–86.

39. Mehdipour, I. Feasibility of Using Near-Field Microwave Reflectometry for Monitoring Autogenous Crack Healing in Cementitious Materials. Cement and Concrete Composites 2018, 13.

40. Mircea, A.C.; Szilagyi, H.; Hegyi, A.; Baera, C. Study of Self-Healing Engineered Cementitious Composites for Durable and Sustainable Infrastructure. Procedia Manufacturing 2020, 46, 871–878, doi:10.1016/j.promfg.2020.05.002.

41. Mu, S.; Schutter, G.D.; Ma, B. Non-Steady State Chloride Diffusion in Concrete with Different Crack Densities. Materials and Structures 2013, 11.

42. Nasim, M. Effect of Crystalline Admixture, Fly Ash, and PVA Fiber on Self-Healing Capacity of Concrete. Materials Today 2020, 6.

43. Nguy, H.H. Self-Healing Properties of Cement-Based and Alkali-Activated Slag-Based Fiber-Reinforced Composites. Construction and Building Materials 2018, 11.

44. Nguyễn, H.H. Autogenous Healing of High Strength Engineered Cementitious Composites (ECC) Using Calcium-Containing Binders. Construction and Building Materials 2020, 16.

45. Nishiwaki, T.; Koda, M. Experimental Study on Self-Healing Capability of FRCC Using Different Types of Synthetic Fibers. 2012, 10, 13.

46. Palin, D.; Jonkers, H.M.; Wiktor, V. Autogenous Healing of Sea-Water Exposed Mortar: Quantification through a Simple and Rapid Permeability Test. Cement and Concrete Research 2016, 7.

47. Park, B. Quantitative Evaluation of Crack Self-Healing in Cement-Based Materials by Absorption Test. Construction and Building Materials 2018, 10.

48. Park, B. Self-Healing Capability of Cementitious Materials with Crystalline Admixtures and Super Absorbent Polymers (SAPs). Construction and Building Materials 2018, 13.

49. Qian, S.; Zhou, J. Self-Healing Behavior of Strain Hardening Cementitious Composites Incorporating Local Waste Materials. 2009, 9.

50. Qiu, J.; He, S.; Yang, E.-H. Polymeric FIber and Hydraulic Cement Matrix. Cement and Concrete Research 2019, 9.

51. Qiu, J. Autogenous Healing of Fiber-Reinforced Reactive Magnesia-Based Tensile Strain-Hardening Composites. Cement and Concrete Research 2019, 13.

52. Qiu, J. Coupled Effects of Crack Width, Slag Content, and Conditioning Alkalinity on Autogenous Healing of Engineered Cementitious Composites. Cement and Concrete Composites 2016, 10.

53. Rajasegar, M. Hybrid Effect of Poly Vinyl Alcohol, Expansive Minerals, Nano-Silica and Rice Husk Ash on the Self-Healing Ability of Concrete. Materials Today 2021, 9.

54. Rauf, M. Comparative Performance of Different Bacteria Immobilized in Natural Fibers for Self-Healing in Concrete. Construction and Building Materials 2020, 13.

55. Reddy, C.M.K. Effect of Crystalline Admixtures, Polymers and Fibers on Self Healing Concrete - a Review. Materials Today 2020, 8.

56. Reddy, T.C.S. Macro Mechanical Properties of Self Healing Concrete with Crystalline Admixture under Different Environments. Ain Shams Engineering Journal 2019, 10.

57. Reinhardt, H.-W.; Jooss, M. Permeability and Self-Healing of Cracked Concrete as a Function of Temperature and Crack Width. Cement and concrete research 2003, 33, 981–985.

58. Roig-Flores, M. Effect of Crystalline Admixtures on the Self-Healing Capability of Early-Age Concrete Studied by Means of Permeability and Crack Closing Tests. Construction and Building Materials 2016, 11.

59. Roig-Flores, M. Self-Healing Capability of Concrete with Crystalline Admixtures in Different Environments. Construction and Building Materials 2015, 11.

60. Ruan, S. Influence of Crack Width on the Stiffness Recovery and Self-Healing of Reactive Magnesia-Based Binders under CO2-H2O Conditioning. Construction and Building Materials 2021, 13.

61. Beshr, B.S.S. Feasibility Assessment on Self-Healing Ability of Cementitious Composites with MgO. Journal of Building Engineering 2021, 8.

62. Shao-feng, Z. Experimental Determination Of Chloride Penetration In Cracked Concrete Beams. 5.

63. Siad, H. Influence of Limestone Powder on Mechanical, Physical and Self-Healing Behavior of Engineered Cementitious Composites. Construction and Building Materials 2015, 10.

64. Sidiq, A. Microstructural Analysis of Healing Efficiency in Highly Durable Concrete. Construction and Building Materials 2019, 15.

65. Singh, H. Influence of Cellulose Fiber Addition on Self-Healing and Water Permeability of Concrete. Case Studies in Construction Materials 2020, 15.

66. Sisomphon, K. Self-Healing of Surface Cracks in Mortars with Expansive Additive and Crystalline Additive. 2012, 9.

67. Snoeck, D.; Van Tittelboom, K.; Steuperaert, S.; Dubruel, P.; De Belie, N. Self-Healing Cementitious Materials by the Combination of Microfibres and Superabsorbent Polymers. Journal of Intelligent Material Systems and Structures 2014, 25, 13–24.

68. Snoeck, D. Mechanical and Self-Healing Properties of Cementitious Composites Reinforced with Flax and Cottonised Flax, and Compared with Polyvinyl Alcohol Fibres. b i o s y s t e m s e n g i n e e r i n g 11.

69. Snoeck, D. Improved Multiple Cracking and Autogenous Healing in Cementitious Materials by Means of Chemically-Treated Natural Fibres. b i o s y s t e m s e n g i n e e r i n g 13.

70. Song, X.F.; Wei, J.F.; He, T.S. A Method to Repair Concrete Leakage through Cracks by Synthesizing Super-Absorbent Resin in Situ. Construction and Building Materials 2009, 6.

71. Su, Y.-F. Autogenous Healing Performance of Internal Curing Agent-Based Self-Healing Cementitious Composite. Cement and Concrete Composites 2020, 8.

72. Suleiman, A.R. Effect of Environmental Exposure on Autogenous Self-Healing of Cracked Cement-Based Materials. Cement and Concrete Research 2018, 12.

73. Tomczak, K.; Jakubowski, J. The Effects of Age, Cement Content, and Healing Time on the Self-Healing Ability of High-Strength Concrete. Construction and Building Materials 2018, 187, 149–159, doi:10.1016/j.conbuildmat.2018.07.176.

74. Tziviloglou, E.; Wiktor, V.; Jonkers, H.M.; Schlangen, E. Bacteria-Based Self-Healing Concrete to Increase Liquid Tightness of Cracks. Construction and Building Materials 2016, 122, 118–125, doi:10.1016/j.conbuildmat.2016.06.080.

75. Van Tittelboom, K.; De Belie, N.; Van Loo, D.; Jacobs, P. Self-Healing Efficiency of Cementitious Materials Containing Tubular Capsules Filled with Healing Agent. Cement and Concrete Composites 2011, 33, 497–505, doi:10.1016/j.cemconcomp.2011.01.004.

76. Van Tittelboom, K.; Wang, J.; Araújo, M.; Snoeck, D.; Gruyaert, E.; Debbaut, B.; Derluyn, H.; Cnudde, V.; Tsangouri, E.; Van Hemelrijck, D.; et al. Comparison of Different Approaches for Self-Healing Concrete in a Large-Scale Lab Test. Construction and Building Materials 2016, 107, 125–137, doi:10.1016/j.conbuildmat.2015.12.186.

77. Wang, R.; Yu, J.; Gu, S.; He, P.; Han, X.; Liu, Q. Investigation of Self-Healing Capability on Surface and Internal Cracks of Cement Mortar with Ion Chelator. Construction and Building Materials 2020, 236, 117598, doi:10.1016/j.conbuildmat.2019.117598.

78. Wang, W.; Liu, J.; Agostini, F.; Davy, C.A.; Skoczylas, F.; Corvez, D. Durability of an Ultra High Performance Fiber Reinforced Concrete (UHPFRC) under Progressive Aging. Cement and Concrete Research 2014, 55, 1–13, doi:10.1016/j.cemconres.2013.09.008.

79. Wang, X.; Huang, Y.; Huang, Y.; Zhang, J.; Fang, C.; Yu, K.; Chen, Q.; Li, T.; Han, R.; Yang, Z.; et al. Laboratory and Field Study on the Performance of Microcapsule-Based Self-Healing Concrete in Tunnel Engineering. Construction and Building Materials 2019, 220, 90–101, doi:10.1016/j.conbuildmat.2019.06.017.

80. Wiktor, V.; Jonkers, H.M. Quantification of Crack-Healing in Novel Bacteria-Based Self-Healing Concrete. Cement and Concrete Composites 2011, 33, 763–770, doi:10.1016/j.cemconcomp.2011.03.012.

81. Win, P.P.; Watanabe, M.; Machida, A. Penetration Profile of Chloride Ion in Cracked Reinforced Concrete. Cement and Concrete Research 2004, 34, 1073–1079, doi:10.1016/j.cemconres.2003.11.020.

82. Wu, M.; Hu, X.; Zhang, Q.; Cheng, W.; Xue, D.; Zhao, Y. Application of Bacterial Spores Coated by a Green Inorganic Cementitious Material for the Self-Healing of Concrete Cracks. Cement and Concrete Composites 2020, 113, 103718, doi:10.1016/j.cemconcomp.2020.103718.

83. Xu, J.; Wang, X. Self-Healing of Concrete Cracks by Use of Bacteria-Containing Low Alkali Cementitious Material. Construction and Building Materials 2018, 167, 1–14, doi:10.1016/j.conbuildmat.2018.02.020.

84. Xue, C.; Li, W.; Qu, F.; Sun, Z.; Shah, S.P. Self-Healing Efficiency and Crack Closure of Smart Cementitious Composite with Crystalline Admixture and Structural Polyurethane. Construction and Building Materials 2020, 260, 119955, doi:10.1016/j.conbuildmat.2020.119955.

85. Xue, C.; Li, W.; Luo, Z.; Wang, K.; Castel, A. Effect of Chloride Ingress on Self-Healing Recovery of Smart Cementitious Composite Incorporating Crystalline Admixture and MgO Expansive Agent. Cement and Concrete Research 2021, 139, 106252, doi:10.1016/j.cemconres.2020.106252.

86. Yang, Y.; Lepech, M.D.; Yang, E.-H.; Li, V.C. Autogenous Healing of Engineered Cementitious Composites under Wet–Dry Cycles. Cement and Concrete Research 2009, 39, 382–390, doi:10.1016/j.cemconres.2009.01.013.

87. Yang, Y.; Yang, E.-H.; Li, V.C. Autogenous Healing of Engineered Cementitious Composites at Early Age. Cement and Concrete Research 2011, 41, 176–183, doi:10.1016/j.cemconres.2010.11.002.

88. Yang, Z.; Hollar, J.; He, X.; Shi, X. Laboratory Assessment of a Self-Healing Cementitious Composite. Transportation Research Record 9.

89. Ye, H.; Jin, N.; Jin, X.; Fu, C. Model of Chloride Penetration into Cracked Concrete Subject to Drying–Wetting Cycles. Construction and Building Materials 2012, 36, 259–269, doi:10.1016/j.conbuildmat.2012.05.027.

90. Yıldırım, G.; Khiavi, A.H.; Yeşilmen, S.; Şahmaran, M. Self-Healing Performance of Aged Cementitious Composites. Cement and Concrete Composites 2018, 87, 172–186, doi:10.1016/j.cemconcomp.2018.01.004.

91. Yildirim, G.; Aras, G.H.; Banyhussan, Q.S.; Şahmaran, M.; Lachemi, M. Estimating the Self-Healing Capability of Cementitious Composites through Non-Destructive Electrical-Based Monitoring. NDT & E International 2015, 76, 26–37, doi:10.1016/j.ndteint.2015.08.005.

92. Yildirim, G.; Sahmaran, M.; Balcikanli, M.; Ozbay, E.; Lachemi, M. Influence of Cracking and Healing on the Gas Permeability of Cementitious Composites. Construction and Building Materials 2015, 85, 217–226, doi:10.1016/j.conbuildmat.2015.02.095.

93. Zha, Y.; Yu, J.; Wang, R.; He, P.; Cao, Z. Effect of Ion Chelating Agent on Self-Healing Performance of Cement-Based Materials. Construction and Building Materials 2018, 190, 308–316, doi:10.1016/j.conbuildmat.2018.09.115.

94. Zhang, L.V.; Suleiman, A.R.; Nehdi, M.L. Self-Healing in Fiber-Reinforced Alkali-Activated Slag Composites Incorporating Different Additives. Construction and Building Materials 2020, 262, 120059, doi:10.1016/j.conbuildmat.2020.120059.

95. Zhang, P.; Dai, Y.; Ding, X.; Zhou, C.; Xue, X.; Zhao, T. Self-Healing Behaviour of Multiple Microcracks of Strain Hardening Cementitious Composites (SHCC). Construction and Building Materials 2018, 169, 705–715, doi:10.1016/j.conbuildmat.2018.03.032.

96. Zhang, Z.; Zhang, Q. Self-Healing Ability of Engineered Cementitious Composites (ECC) under Different Exposure Environments. Construction and Building Materials 2017, 156, 142–151, doi:10.1016/j.conbuildmat.2017.08.166.

97. Zhang, Z.; Qian, S.; Ma, H. Investigating Mechanical Properties and Self-Healing Behavior of Micro-Cracked ECC with Different Volume of Fly Ash. Construction and Building Materials 2014, 52, 17–23, doi:10.1016/j.conbuildmat.2013.11.001.

98. Zhao, G.; Li, J.; Shi, M.; Cui, J.; Xie, F. Degradation of Cast-in-Situ Concrete Subjected to Sulphate-Chloride Combined Attack. Construction and Building Materials 2020, 241, 117995, doi:10.1016/j.conbuildmat.2019.117995.

99. Zhong, W.; Yao, W. Influence of Damage Degree on Self-Healing of Concrete. Construction and Building Materials 2008, 22, 1137–1142, doi:10.1016/j.conbuildmat.2007.02.006.

100. Zhu, H.; Zhang, D.; Wang, T.; Wu, H.; Li, V.C. Mechanical and Self-Healing Behavior of Low Carbon Engineered Cementitious Composites Reinforced with PP-Fibers. Construction and Building Materials 2020, 259, 119805, doi:10.1016/j.conbuildmat.2020.119805.

The additional 136 articles that are not used due to restraints of required data in this study. However, with more additional scope of parameters, they can be used in future studies.

1. Akhavan, A.; Shafaatian, S.-M.-H.; Rajabipour, F. Quantifying the Effects of Crack Width, Tortuosity, and Roughness on Water Permeability of Cracked Mortars. Cement and Concrete Research 2012, 42, 313–320, doi:10.1016/j.cemconres.2011.10.002.
2. Al-Ansari, M.; Abu-Taqa, A.G.; Hassan, M.M.; Senouci, A.; Milla, J. Performance of Modified Self-Healing Concrete with Calcium Nitrate Microencapsulation. Construction and Building Materials 2017, 149, 525–534, doi:10.1016/j.conbuildmat.2017.05.152.
3. Alazhari, M.; Sharma, T.; Heath, A.; Cooper, R.; Paine, K. Application of Expanded Perlite Encapsulated Bacteria and Growth Media for Self-Healing Concrete. Construction and Building Materials 2018, 160, 610–619, doi:10.1016/j.conbuildmat.2017.11.086.
4. Algaifi, H.A.; Bakar, S.A.; Sam, A.R.Mohd.; Abidin, A.R.Z.; Shahir, S.; AL-Towayti, W.A.H. Numerical Modeling for Crack Self-Healing Concrete by Microbial Calcium Carbonate. Construction and Building Materials 2018, 189, 816–824, doi:10.1016/j.conbuildmat.2018.08.218.
5. Alghamri, R.; Kanellopoulos, A.; Al-Tabbaa, A. Impregnation and Encapsulation of Lightweight Aggregates for Self-Healing Concrete. Construction and Building Materials 2016, 124, 910–921, doi:10.1016/j.conbuildmat.2016.07.143.
6. Alghamri, R.; Kanellopoulos, A.; Litina, C.; Al-Tabbaa, A. Preparation and Polymeric Encapsulation of Powder Mineral Pellets for Self-Healing Cement Based Materials. Construction and Building Materials 2018, 186, 247–262, doi:10.1016/j.conbuildmat.2018.07.128.
7. Aliko-Benítez, A.; Doblaré, M.; Sanz-Herrera, J.A. Chemical-Diffusive Modeling of the Self-Healing Behavior in Concrete. International Journal of Solids and Structures 2015, 69–70, 392–402, doi:10.1016/j.ijsolstr.2015.05.011.
8. Al-Salloum, Y.; Hadi, S.; Abbas, H.; Almusallam, T.; Moslem, M.A. Bio-Induction and Bioremediation of Cementitious Composites Using Microbial Mineral Precipitation – A Review. Construction and Building Materials 2017, 154, 857–876, doi:10.1016/j.conbuildmat.2017.07.203.

9. Bentur, A.; Mitchell, D. Material Performance Lessons. Cement and Concrete Research 2008, 38, 259–272, doi:10.1016/j.cemconres.2007.09.009.

10. Berrocal, C.G.; Lundgren, K.; Löfgren, I. Corrosion of Steel Bars Embedded in Fibre Reinforced Concrete under Chloride Attack: State of the Art. Cement and Concrete Research 2016, 80, 69–85, doi:10.1016/j.cemconres.2015.10.006.

11. Bhaskar, S.; Anwar Hossain, K.M.; Lachemi, M.; Wolfaardt, G.; Otini Kroukamp, M. Effect of Self-Healing on Strength and Durability of Zeolite-Immobilized Bacterial Cementitious Mortar Composites. Cement and Concrete Composites 2017, 82, 23–33, doi:10.1016/j.cemconcomp.2017.05.013.

12. Boulay, C.; Dal Pont, S.; Belin, P. Real-Time Evolution of Electrical Resistance in Cracking Concrete. Cement and Concrete Research 2009, 39, 825–831, doi:10.1016/j.cemconres.2009.06.003.

13. Byoungsun, P.; Young, C.C. Investigating a New Method to Assess the Self-Healing Performance of Hardened Cement Pastes Containing Supplementary Cementitious Materials and Crystalline Admixtures. Journal of Materials Research and Technology 2019, 8, 6058–6073, doi:10.1016/j.jmrt.2019.09.080.

14. Caggiano, A.; Etse, G.; Ferrara, L.; Krelani, V. Zero-Thickness Interface Constitutive Theory for Concrete Self-Healing Effects. Computers & Structures 2017, 186, 22–34, doi:10.1016/j.compstruc.2017.02.005.

15. Chandra Sekhara Reddy, T.; Ravitheja, A. Macro Mechanical Properties of Self Healing Concrete with Crystalline Admixture under Different Environments. Ain Shams Engineering Journal 2019, 10, 23–32, doi:10.1016/j.asej.2018.01.005.

16. Charron, J.-P.; Denarié, E.; Brühwiler, E. Transport Properties of Water and Glycol in an Ultra High Performance Fiber Reinforced Concrete (UHPFRC) under High Tensile Deformation. Cement and Concrete Research 2008, 38, 689–698, doi:10.1016/j.cemconres.2007.12.006.

17. Chen, J.; Ye, G. A Lattice Boltzmann Single Component Model for Simulation of the Autogenous Self-Healing Caused by Further Hydration in Cementitious Material at Mesoscale. Cement and Concrete Research 2019, 123, 105782, doi:10.1016/j.cemconres.2019.105782.

18. Chitez, A.S.; Jefferson, A.D. A Coupled Thermo-Hygro-Chemical Model for Characterising Autogenous Healing in Ordinary Cementitious Materials. Cement and Concrete Research 2016, 88, 184–197, doi:10.1016/j.cemconres.2016.07.002.

19. Choi, S.-G.; Wang, K.; Wen, Z.; Chu, J. Mortar Crack Repair Using Microbial Induced Calcite Precipitation Method. Cement and Concrete Composites 2017, 83, 209–221, doi:10.1016/j.cemconcomp.2017.07.013.

20. Choi, Y.C.; Park, B. Enhanced Autogenous Healing of Ground Granulated Blast Furnace Slag Blended Cements and Mortars. Journal of Materials Research and Technology 2019, 8, 3443–3452, doi:10.1016/j.jmrt.2019.06.010.

21. Cuenca, E.; Tejedor, A.; Ferrara, L. A Methodology to Assess Crack-Sealing Effectiveness of Crystalline Admixtures under Repeated Cracking-Healing Cycles. Construction and Building Materials 2018, 179, 619–632, doi:10.1016/j.conbuildmat.2018.05.261.

22. Da Silva, F.B.; De Belie, N.; Boon, N.; Verstraete, W. Production of Non-Axenic Ureolytic Spores for Self-Healing Concrete Applications. Construction and Building Materials 2015, 93, 1034–1041, doi:10.1016/j.conbuildmat.2015.05.049.

23. De Nardi, C.; Cecchi, A.; Ferrara, L.; Benedetti, A.; Cristofori, D. Effect of Age and Level of Damage on the Autogenous Healing of Lime Mortars. Composites Part B: Engineering 2017, 124, 144–157, doi:10.1016/j.compositesb.2017.05.041.

24. Di Luzio, G.; Ferrara, L.; Krelani, V. Numerical Modeling of Mechanical Regain Due to Self-Healing in Cement Based Composites. Cement and Concrete Composites 2018, 86, 190–205, doi:10.1016/j.cemconcomp.2017.11.006.

25. Dolado, J.S.; van Breugel, K. Recent Advances in Modeling for Cementitious Materials. Cement and Concrete Research 2011, 41, 711–726, doi:10.1016/j.cemconres.2011.03.014.

26. Dong, B.; Fang, G.; Wang, Y.; Liu, Y.; Hong, S.; Zhang, J.; Lin, S.; Xing, F. Performance Recovery Concerning the Permeability of Concrete by Means of a Microcapsule Based Self-Healing System. Cement and Concrete Composites 2017, 78, 84–96, doi:10.1016/j.cemconcomp.2016.12.005.

27. Dorozhkin, S.V. Biphasic, Triphasic and Multiphasic Calcium Orthophosphates. Acta Biomaterialia 2012, 8, 963–977, doi:10.1016/j.actbio.2011.09.003.

28. Ducasse-Lapeyrusse, J.; Gagné, R.; Lors, C.; Damidot, D. Effect of Calcium Gluconate, Calcium Lactate, and Urea on the Kinetics of Self-Healing in Mortars. Construction and Building Materials 2017, 157, 489–497, doi:10.1016/j.conbuildmat.2017.09.115.

29. Fan, S.; Li, X.; Li, M. The Effects of Damage and Self-Healing on Impedance Spectroscopy of Strain-Hardening Cementitious Materials. Cement and Concrete Research 2018, 106, 77–90, doi:10.1016/j.cemconres.2018.01.016.

30. Fang, G.; Liu, Y.; Qin, S.; Ding, W.; Zhang, J.; Hong, S.; Xing, F.; Dong, B. Visualized Tracing of Crack Self-Healing Features in Cement/Microcapsule System with X-Ray Microcomputed Tomography. Construction and Building Materials 2018, 179, 336–347, doi:10.1016/j.conbuildmat.2018.05.193.

31. Feiteira, J.; Gruyaert, E.; De Belie, N. Self-Healing of Moving Cracks in Concrete by Means of Encapsulated Polymer Precursors. Construction and Building Materials 2016, 102, 671–678, doi:10.1016/j.conbuildmat.2015.10.192.

32. Feiteira, J.; Tsangouri, E.; Gruyaert, E.; Lors, C.; Louis, G.; De Belie, N. Monitoring Crack Movement in Polymer-Based Self-Healing Concrete through Digital Image Correlation, Acoustic Emission Analysis and SEM in-Situ Loading. Materials & Design 2017, 115, 238–246, doi:10.1016/j.matdes.2016.11.050.

33. Feng, J.; Su, Y.; Qian, C. Coupled Effect of PP Fiber, PVA Fiber and Bacteria on Self-Healing Efficiency of Early-Age Cracks in Concrete. Construction and Building Materials 2019, 228, 116810, doi:10.1016/j.conbuildmat.2019.116810.

34. Ferraresi, R.; Palloshi, A.; Aprigliano, G.; Caravaggi, C.; Centola, M.; Sozzi, F.; Danzi, G.B.; Manzi, M. Angioplasty of Below-the-Elbow Arteries in Critical Hand Ischaemia. European Journal of Vascular and Endovascular Surgery 2012, 43, 73–80, doi:10.1016/j.ejvs.2011.10.006.

35. Galan, I.; Baldermann, A.; Kusterle, W.; Dietzel, M.; Mittermayr, F. Durability of Shotcrete for Underground Support– Review and Update. Construction and Building Materials 2019, 202, 465–493, doi:10.1016/j.conbuildmat.2018.12.151.

36. García Calvo, J.L.; Pérez, G.; Carballosa, P.; Erkizia, E.; Gaitero, J.J.; Guerrero, A. Development of Ultra-High Performance Concretes with Self-Healing Micro/Nano-Additions. Construction and Building Materials 2017, 138, 306–315, doi:10.1016/j.conbuildmat.2017.02.015.

37. García Calvo, J.L.; Pérez, G.; Carballosa, P.; Erkizia, E.; Gaitero, J.J.; Guerrero, A. 3 - The effect of nanoparticles on the self-healing capacity of high performance concrete. In Nanotechnology in Eco-efficient Construction (Second Edition); Pacheco-Torgal, F., Diamanti, M.V., Nazari, A., Granqvist, C.G., Pruna, A., Amirkhanian, S., Eds.; Woodhead Publishing Series in Civil and Structural Engineering; Woodhead Publishing, 2019; pp. 43–67 ISBN 978-0-08-102641-0.

38. Ghantous, R.M.; Poyet, S.; L'Hostis, V.; Tran, N.-C.; François, R. Effect of Crack Openings on Carbonation-Induced Corrosion. Cement and Concrete Research 2017, 95, 257–269, doi:10.1016/j.cemconres.2017.02.014.

39. Gupta, S.; Kua, H.W.; Pang, S.D. Healing Cement Mortar by Immobilization of Bacteria in Biochar: An Integrated Approach of Self-Healing and Carbon Sequestration. Cement and Concrete Composites 2018, 86, 238–254, doi:10.1016/j.cemconcomp.2017.11.015.

40. Gupta, S.; Pang, S.D.; Kua, H.W. Autonomous Healing in Concrete by Bio-Based Healing Agents – A Review. Construction and Building Materials 2017, 146, 419–428, doi:10.1016/j.conbuildmat.2017.04.111.

41. He, J.; Shi, X. Developing an Abiotic Capsule-Based Self-Healing System for Cementitious Materials: The State of Knowledge. Construction and Building Materials 2017, 156, 1096–1113, doi:10.1016/j.conbuildmat.2017.09.041.

42. He, Z.; Shen, A.; Guo, Y.; Lyu, Z.; Li, D.; Qin, X.; Zhao, M.; Wang, Z. Cement-Based Materials Modified with Superabsorbent Polymers: A Review. Construction and Building Materials 2019, 225, 569–590, doi:10.1016/j.conbuildmat.2019.07.139.

43. Hilloulin, B.; Grondin, F.; Matallah, M.; Loukili, A. Modelling of Autogenous Healing in Ultra High Performance Concrete. Cement and Concrete Research 2014, 61–62, 64–70, doi:10.1016/j.cemconres.2014.04.003.

44. Hilloulin, B.; Legland, J.-B.; Lys, E.; Abraham, O.; Loukili, A.; Grondin, F.; Durand, O.; Tournat, V. Monitoring of Autogenous Crack Healing in Cementitious Materials by the Nonlinear Modulation of Ultrasonic Coda Waves, 3D Microscopy and X-Ray Microtomography. Construction and Building Materials 2016, 123, 143–152, doi:10.1016/j.conbuildmat.2016.06.138.

45. Hoseini, M.; Bindiganavile, V.; Banthia, N. The Effect of Mechanical Stress on Permeability of Concrete: A Review. Cement and Concrete Composites 2009, 31, 213–220, doi:10.1016/j.cemconcomp.2009.02.003.

46. Huang, H.; Ye, G.; Qian, C.; Schlangen, E. Self-Healing in Cementitious Materials: Materials, Methods and Service Conditions. Materials & Design 2016, 92, 499–511, doi:10.1016/j.matdes.2015.12.091.

47. Huseien, G.F.; Shah, K.W.; Sam, A.R.M. Sustainability of Nanomaterials Based Self-Healing Concrete: An All-Inclusive Insight. Journal of Building Engineering 2019, 23, 155–171, doi:10.1016/j.jobe.2019.01.032.

48. In, C.-W.; Holland, R.B.; Kim, J.-Y.; Kurtis, K.E.; Kahn, L.F.; Jacobs, L.J. Monitoring and Evaluation of Self-Healing in Concrete Using Diffuse Ultrasound. NDT & E International 2013, 57, 36–44, doi:10.1016/j.ndteint.2013.03.005.

49. Ismail, M.; Toumi, A.; François, R.; Gagné, R. Effect of Crack Opening on the Local Diffusion of Chloride in Cracked Mortar Samples. Cement and Concrete Research 2008, 38, 1106–1111, doi:10.1016/j.cemconres.2008.03.009.

50. Ismail, M.; Toumi, A.; François, R.; Gagné, R. Effect of Crack Opening on the Local Diffusion of Chloride in Cracked Mortar Samples. Cement and Concrete Research 2008, 38, 1106–1111, doi:10.1016/j.cemconres.2008.03.009.

51. Jiang, J.; Zheng, X.; Wu, S.; Liu, Z.; Zheng, Q. Nondestructive Experimental Characterization and Numerical Simulation on Self-Healing and Chloride Ion Transport in Cracked Ultra-High Performance Concrete. Construction and Building Materials 2019, 198, 696–709, doi:10.1016/j.conbuildmat.2018.11.054.

52. Jiang, J.; Zheng, X.; Wu, S.; Liu, Z.; Zheng, Q. Nondestructive Experimental Characterization and Numerical Simulation on Self-Healing and Chloride Ion Transport in Cracked Ultra-High Performance Concrete. Construction and Building Materials 2019, 198, 696–709, doi:10.1016/j.conbuildmat.2018.11.054.

53. Kalhori, H.; Bagherpour, R. Application of Carbonate Precipitating Bacteria for Improving Properties and Repairing Cracks of Shotcrete. Construction and Building Materials 2017, 148, 249–260, doi:10.1016/j.conbuildmat.2017.05.074.

54. Kewalramani, M.A.; Mohamed, O.A.; Syed, Z.I. Engineered Cementitious Composites for Modern Civil Engineering Structures in Hot Arid Coastal Climatic Conditions. Procedia Engineering 2017, 180, 767–774, doi:10.1016/j.proeng.2017.04.237.

55. Kua, H.W.; Gupta, S.; Aday, A.N.; Srubar, W.V. Biochar-Immobilized Bacteria and Superabsorbent Polymers Enable Self-Healing of Fiber-Reinforced Concrete after Multiple Damage Cycles. Cement and Concrete Composites 2019, 100, 35–52, doi:10.1016/j.cemconcomp.2019.03.017.

56. Lepech, M.D.; Li, V.C. Water Permeability of Engineered Cementitious Composites. Cement and Concrete Composites 2009, 31, 744–753, doi:10.1016/j.cemconcomp.2009.07.002.

57. Lepech, M.D.; Li, V.C. Water Permeability of Engineered Cementitious Composites. Cement and Concrete Composites 2009, 31, 744–753, doi:10.1016/j.cemconcomp.2009.07.002.

58. Li, Q.; Liu, Z.; Chen, W.; Yuan, B.; Liu, X.; Chen, W. A Novel Bio-Inspired Bone-Mimic Self-Healing Cement Paste Based on Hydroxyapatite Formation. Cement and Concrete Composites 2019, 104, 103357, doi:10.1016/j.cemconcomp.2019.103357.

59. Li, V.C. High-Performance and Multifunctional Cement-Based Composite Material. Engineering 2019, 5, 250–260, doi:10.1016/j.eng.2018.11.031.

60. Liu, K.; Shui, Z.; Sun, T.; Ling, G.; Li, X.; Cheng, S. Effects of Combined Expansive Agents and Supplementary Cementitious Materials on the Mechanical Properties, Shrinkage and Chloride Penetration of Self-Compacting Concrete. Construction and Building Materials 2019, 211, 120–129, doi:10.1016/j.conbuildmat.2019.03.143.

61. Lors, C.; Ducasse-Lapeyrusse, J.; Gagné, R.; Damidot, D. Microbiologically Induced Calcium Carbonate Precipitation to Repair Microcracks Remaining after Autogenous Healing of Mortars. Construction and Building Materials 2017, 141, 461–469, doi:10.1016/j.conbuildmat.2017.03.026.

62. Luo, J.; Chen, X.; Crump, J.; Zhou, H.; Davies, D.G.; Zhou, G.; Zhang, N.; Jin, C. Interactions of Fungi with Concrete: Significant Importance for Bio-Based Self-Healing Concrete. Construction and Building Materials 2018, 164, 275–285, doi:10.1016/j.conbuildmat.2017.12.233.

63. Ma, H.; Zhang, Z.; Ding, B.; Tu, X. Investigation on the Adhesive Characteristics of Engineered Cementitious Composites (ECC) to Steel Bridge Deck. Construction and Building Materials 2018, 191, 679–691, doi:10.1016/j.conbuildmat.2018.10.056.

64. Maes, M.; Snoeck, D.; De Belie, N. Chloride Penetration in Cracked Mortar and the Influence of Autogenous Crack Healing. Construction and Building Materials 2016, 115, 114–124, doi:10.1016/j.conbuildmat.2016.03.180.

65. Maes, M.; Van Tittelboom, K.; De Belie, N. The Efficiency of Self-Healing Cementitious Materials by Means of Encapsulated Polyurethane in Chloride Containing Environments. Construction and Building Materials 2014, 71, 528–537, doi:10.1016/j.conbuildmat.2014.08.053.

66. Mamun, M.; Bindiganavile, V. Sulphate Resistance of Fibre Reinforced Cement-Based Foams. Construction and Building Materials 2011, 25, 3427–3442, doi:10.1016/j.conbuildmat.2011.03.034.

67. Mangadlao, J.D.; Cao, P.; Advincula, R.C. Smart Cements and Cement Additives for Oil and Gas Operations. Journal of Petroleum Science and Engineering 2015, 129, 63–76, doi:10.1016/j.petrol.2015.02.009.

68. Mechtcherine, V. Towards a Durability Framework for Structural Elements and Structures Made of or Strengthened with High-Performance Fibre-Reinforced Composites. Construction and Building Materials 2012, 31, 94–104, doi:10.1016/j.conbuildmat.2011.12.072.

69. Mechtcherine, V. Towards a Durability Framework for Structural Elements and Structures Made of or Strengthened with High-Performance Fibre-Reinforced Composites. Construction and Building Materials 2012, 31, 94–104, doi:10.1016/j.conbuildmat.2011.12.072.

70. Milla, J.; Hassan, M.M.; Rupnow, T.; Daly, W.H. Measuring the Crack-Repair Efficiency of Steel Fiber Reinforced Concrete Beams with Microencapsulated Calcium Nitrate. Construction and Building Materials 2019, 201, 526–538, doi:10.1016/j.conbuildmat.2018.12.193.

71. Mondal, S.; Ghosh, A. (Dey) Investigation into the Optimal Bacterial Concentration for Compressive Strength Enhancement of Microbial Concrete. Construction and Building Materials 2018, 183, 202–214, doi:10.1016/j.conbuildmat.2018.06.176.

72. Morin, V.; Cohen Tenoudji, F.; Feylessoufi, A.; Richard, P. Superplasticizer Effects on Setting and Structuration Mechanisms of Ultrahigh-Performance Concrete. Cement and Concrete Research 2001, 31, 63–71, doi:10.1016/S0008-8846(00)00428-2.

73. Mors, R.M.; Jonkers, H.M. Feasibility of Lactate Derivative Based Agent as Additive for Concrete for Regain of Crack Water Tightness by Bacterial Metabolism. Industrial Crops and Products 2017, 106, 97–104, doi:10.1016/j.indcrop.2016.10.037.

74. Muhammad, N.Z.; Shafaghat, A.; Keyvanfar, A.; Abd. Majid, M.Z.; Ghoshal, S.K.; Mohammadyan Yasouj, S.E.; Ganiyu, A.A.; Samadi Kouchaksaraei, M.; Kamyab, H.; Taheri, M.M.; et al. Tests and Methods of Evaluating the Self-Healing Efficiency of Concrete: A Review. Construction and Building Materials 2016, 112, 1123–1132, doi:10.1016/j.conbuildmat.2016.03.017.

75. Nguyễn, H.H.; Choi, J.-I.; Kim, H.-K.; Lee, B.Y. Effects of the Type of Activator on the Self-Healing Ability of Fiber-Reinforced Alkali-Activated Slag-Based Composites at an Early Age. Construction and Building Materials 2019, 224, 980–994, doi:10.1016/j.conbuildmat.2019.07.113.

76. Nguyen, T.H.; Ghorbel, E.; Fares, H.; Cousture, A. Bacterial Self-Healing of Concrete and Durability Assessment. Cement and Concrete Composites 2019, 104, 103340, doi:10.1016/j.cemconcomp.2019.103340.

77. Oucif, C.; Voyiadjis, G.Z.; Rabczuk, T. Modeling of Damage-Healing and Nonlinear Self-Healing Concrete Behavior: Application to Coupled and Uncoupled Self-Healing Mechanisms. Theoretical and Applied Fracture Mechanics 2018, 96, 216–230, doi:10.1016/j.tafmec.2018.04.010.

78. Pal Kaur, N.; Kumar Shah, J.; Majhi, S.; Mukherjee, A. Healing and Simultaneous Ultrasonic Monitoring of Cracks in Concrete. Materials Today Communications 2019, 18, 87–99, doi:10.1016/j.mtcomm.2018.10.022.

79. Palin, D.; Wiktor, V.; Jonkers, H.M. Autogenous Healing of Marine Exposed Concrete: Characterization and Quantification through Visual Crack Closure. Cement and Concrete Research 2015, 73, 17–24, doi:10.1016/j.cemconres.2015.02.021.

80. Pang, B.; Zhou, Z.; Hou, P.; Du, P.; Zhang, L.; Xu, H. Autogenous and Engineered Healing Mechanisms of Carbonated Steel Slag Aggregate in Concrete. Construction and Building Materials 2016, 107, 191–202, doi:10.1016/j.conbuildmat.2015.12.191.

81. Phung, Q.T.; Maes, N.; De Schutter, G.; Jacques, D.; Ye, G. Determination of Water Permeability of Cementitious Materials Using a Controlled Constant Flow Method. Construction and Building Materials 2013, 47, 1488–1496, doi:10.1016/j.conbuildmat.2013.06.074.

82. Qiu, J.; He, S.; Yang, E.-H. Autogenous Healing and Its Enhancement of Interface between Micro Polymeric Fiber and Hydraulic Cement Matrix. Cement and Concrete Research 2019, 124, 105830, doi:10.1016/j.cemconres.2019.105830.

83. Qureshi, T.; Kanellopoulos, A.; Al-Tabbaa, A. Autogenous Self-Healing of Cement with Expansive Minerals-I: Impact in Early Age Crack Healing. Construction and Building Materials 2018, 192, 768–784, doi:10.1016/j.conbuildmat.2018.10.143.

84. Qureshi, T.; Kanellopoulos, A.; Al-Tabbaa, A. Autogenous Self-Healing of Cement with Expansive Minerals-II: Impact of Age and the Role of Optimised Expansive Minerals in Healing Performance. Construction and Building Materials 2019, 194, 266–275, doi:10.1016/j.conbuildmat.2018.11.027.

85. Qureshi, T.S.; Kanellopoulos, A.; Al-Tabbaa, A. Encapsulation of Expansive Powder Minerals within a Concentric Glass Capsule System for Self-Healing Concrete. Construction and Building Materials 2016, 121, 629–643, doi:10.1016/j.conbuildmat.2016.06.030.

86. Rahal, S.; Sellier, A. Influence of Crack Reclosure on Concrete Permeability. Theoretical and Applied Fracture Mechanics 2019, 100, 65–77, doi:10.1016/j.tafmec.2018.11.010.

87. Rodríguez, C.R.; Figueiredo, S.C.; Deprez, M.; Snoeck, D.; Schlangen, E.; Šavija, B. Numerical Investigation of Crack Self-Sealing in Cement-Based Composites with Superabsorbent Polymers. Cement and Concrete Composites 2019, 104, 103395, doi:10.1016/j.cemconcomp.2019.103395.

88. Rossi, P.; Tailhan, J.-L.; Le Maou, F.; Gaillet, L.; Martin, E. Basic Creep Behavior of Concretes Investigation of the Physical Mechanisms by Using Acoustic Emission. Cement and Concrete Research 2012, 42, 61–73, doi:10.1016/j.cemconres.2011.07.011.

89. Şahmaran, M.; Keskin, S.B.; Ozerkan, G.; Yaman, I.O. Self-Healing of Mechanically-Loaded Self Consolidating Concretes with High Volumes of Fly Ash. Cement and Concrete Composites 2008, 30, 872–879, doi:10.1016/j.cemconcomp.2008.07.001.

90. Şahmaran, M.; Keskin, S.B.; Ozerkan, G.; Yaman, I.O. Self-Healing of Mechanically-Loaded Self Consolidating Concretes with High Volumes of Fly Ash. Cement and Concrete Composites 2008, 30, 872–879, doi:10.1016/j.cemconcomp.2008.07.001.

91. Şahmaran, M.; Lachemi, M.; Hossain, K.M.A.; Li, V.C. Internal Curing of Engineered Cementitious Composites for Prevention of Early Age Autogenous Shrinkage Cracking. Cement and Concrete Research 2009, 39, 893–901, doi:10.1016/j.cemconres.2009.07.006.

92. Şahmaran, M.; Li, V.C. Durability of Mechanically Loaded Engineered Cementitious Composites under Highly Alkaline Environments. Cement and Concrete Composites 2008, 30, 72–81, doi:10.1016/j.cemconcomp.2007.09.004.

93. Sahmaran, M.; Yildirim, G.; Erdem, T.K. Self-Healing Capability of Cementitious Composites Incorporating Different Supplementary Cementitious Materials. Cement and Concrete Composites 2013, 35, 89–101, doi:10.1016/j.cemconcomp.2012.08.013.

94. Sakulich, A.R. Reinforced Geopolymer Composites for Enhanced Material Greenness and Durability. Sustainable Cities and Society 2011, 1, 195–210, doi:10.1016/j.scs.2011.07.009.

95. Sánchez, M.; Faria, P.; Ferrara, L.; Horszczaruk, E.; Jonkers, H.M.; Kwiecień, A.; Mosa, J.; Peled, A.; Pereira, A.S.; Snoeck, D.; et al. External Treatments for the Preventive Repair of Existing Constructions: A Review. Construction and Building Materials 2018, 193, 435–452, doi:10.1016/j.conbuildmat.2018.10.173.

96. Schlangen, E.; Sangadji, S. Addressing Infrastructure Durability and Sustainability by Self Healing Mechanisms - Recent Advances in Self Healing Concrete and Asphalt. Procedia Engineering 2013, 54, 39–57, doi:10.1016/j.proeng.2013.03.005.

97. Schreiberová, H.; Bílý, P.; Fládr, J.; Šeps, K.; Chylík, R.; Trtík, T. Impact of the Self-Healing Agent Composition on Material Characteristics of Bio-Based Self-Healing Concrete. Case Studies in Construction Materials 2019, 11, e00250, doi:10.1016/j.cscm.2019.e00250.

98. Shaheen, N.; Khushnood, R.A.; Khaliq, W.; Murtaza, H.; Iqbal, R.; Khan, M.H. Synthesis and Characterization of Bio-Immobilized Nano/Micro Inert and Reactive Additives for Feasibility Investigation in Self-Healing Concrete. Construction and Building Materials 2019, 226, 492–506, doi:10.1016/j.conbuildmat.2019.07.202.

99. Sherir, M.A.A.; Hossain, K.M.A.; Lachemi, M. Fresh State, Mechanical & Durability Properties of Strain Hardening Cementitious Composite Produced with Locally Available Aggregates and High Volume of Fly Ash. Construction and Building Materials 2018, 189, 253–264, doi:10.1016/j.conbuildmat.2018.08.204.

100. Sidiq, A.; Gravina, R.; Giustozzi, F. Is Concrete Healing Really Efficient? A Review. Construction and Building Materials 2019, 205, 257–273, doi:10.1016/j.conbuildmat.2019.02.002.

101. Sisomphon, K.; Çopuroğlu, O.; Fraaij, A.L.A. Durability of Blast-Furnace Slag Mortars Subjected to Sodium Monofluorophosphate Application. Construction and Building Materials 2011, 25, 823–828, doi:10.1016/j.conbuildmat.2009.09.010.

102. Snoeck, D.; De Belie, N. From Straw in Bricks to Modern Use of Microfibers in Cementitious Composites for Improved Autogenous Healing – A Review. Construction and Building Materials 2015, 95, 774–787, doi:10.1016/j.conbuildmat.2015.07.018.

103. Snoeck, D.; De Schryver, T.; De Belie, N. Enhanced Impact Energy Absorption in Self-Healing Strain-Hardening Cementitious Materials with Superabsorbent Polymers. Construction and Building Materials 2018, 191, 13–22, doi:10.1016/j.conbuildmat.2018.10.015.

104. Snoeck, D.; Dewanckele, J.; Cnudde, V.; De Belie, N. X-Ray Computed Microtomography to Study Autogenous Healing of Cementitious Materials Promoted by Superabsorbent Polymers. Cement and Concrete Composites 2016, 65, 83–93, doi:10.1016/j.cemconcomp.2015.10.016.

105. Snoeck, D.; Van den Heede, P.; Van Mullem, T.; De Belie, N. Water Penetration through Cracks in Self-Healing Cementitious Materials with Superabsorbent Polymers Studied by Neutron Radiography. Cement and Concrete Research 2018, 113, 86–98, doi:10.1016/j.cemconres.2018.07.002.

106. Somarathna, H.M.C.C.; Raman, S.N.; Mohotti, D.; Mutalib, A.A.; Badri, K.H. The Use of Polyurethane for Structural and Infrastructural Engineering Applications: A State-of-the-Art Review. Construction and Building Materials 2018, 190, 995–1014, doi:10.1016/j.conbuildmat.2018.09.166.

107. Souza, L.; Souza, L.; Silva, F. Autogenous Healing Capability of Natural Curauá Textile Reinforced Concrete. Procedia Engineering 2017, 200, 290–294, doi:10.1016/j.proeng.2017.07.041.

108. Su, Y.; Feng, J.; Jin, P.; Qian, C. Influence of Bacterial Self-Healing Agent on Early Age Performance of Cement-Based Materials. Construction and Building Materials 2019, 218, 224–234, doi:10.1016/j.conbuildmat.2019.05.077.

109. Tam, C.M.; Tam, V.W.Y.; Ng, K.M. Assessing Drying Shrinkage and Water Permeability of Reactive Powder Concrete Produced in Hong Kong. Construction and Building Materials 2012, 26, 79–89, doi:10.1016/j.conbuildmat.2011.05.006.

110. Tang, W.; Kardani, O.; Cui, H. Robust Evaluation of Self-Healing Efficiency in Cementitious Materials – A Review. Construction and Building Materials 2015, 81, 233–247, doi:10.1016/j.conbuildmat.2015.02.054.

111. Tsangouri, E.; Gilabert, F.A.; De Belie, N.; Van Hemelrijck, D.; Zhu, X.; Aggelis, D.G. Concrete Fracture Toughness Increase by Embedding Self-Healing Capsules Using an Integrated Experimental Approach. Construction and Building Materials 2019, 218, 424–433, doi:10.1016/j.conbuildmat.2019.05.138.

112. Van den Heede, P.; Maes, M.; De Belie, N. Influence of Active Crack Width Control on the Chloride Penetration Resistance and Global Warming Potential of Slabs Made with Fly Ash+silica Fume Concrete. Construction and Building Materials 2014, 67, 74–80, doi:10.1016/j.conbuildmat.2013.10.032.

113. Van den Heede, P.; Mignon, A.; Habert, G.; De Belie, N. Cradle-to-Gate Life Cycle Assessment of Self-Healing Engineered Cementitious Composite with in-House Developed (Semi-)Synthetic Superabsorbent Polymers. Cement and Concrete Composites 2018, 94, 166–180, doi:10.1016/j.cemconcomp.2018.08.017.

114. Van den Heede, P.; Mignon, A.; Habert, G.; De Belie, N. Cradle-to-Gate Life Cycle Assessment of Self-Healing Engineered Cementitious Composite with in-House Developed (Semi-)Synthetic Superabsorbent Polymers. Cement and Concrete Composites 2018, 94, 166–180, doi:10.1016/j.cemconcomp.2018.08.017.

115. Van Mullem, T.; Gruyaert, E.; Debbaut, B.; Caspeele, R.; De Belie, N. Novel Active Crack Width Control Technique to Reduce the Variation on Water Permeability Results for Self-Healing Concrete. Construction and Building Materials 2019, 203, 541–551, doi:10.1016/j.conbuildmat.2019.01.105.

116. Vijay, K.; Murmu, M.; Deo, S.V. Bacteria Based Self Healing Concrete – A Review. Construction and Building Materials 2017, 152, 1008–1014, doi:10.1016/j.conbuildmat.2017.07.040.

117. Wang, J.; Dewanckele, J.; Cnudde, V.; Van Vlierberghe, S.; Verstraete, W.; De Belie, N. X-Ray Computed Tomography Proof of Bacterial-Based Self-Healing in Concrete. Cement and Concrete Composites 2014, 53, 289–304, doi:10.1016/j.cemconcomp.2014.07.014.

118. Wang, J.; Mignon, A.; Trenson, G.; Van Vlierberghe, S.; Boon, N.; De Belie, N. A Chitosan Based PH-Responsive Hydrogel for Encapsulation of Bacteria for Self-Sealing Concrete. Cement and Concrete Composites 2018, 93, 309–322, doi:10.1016/j.cemconcomp.2018.08.007.

119. Wang, J.Y.; Snoeck, D.; Van Vlierberghe, S.; Verstraete, W.; De Belie, N. Application of Hydrogel Encapsulated Carbonate Precipitating Bacteria for Approaching a Realistic Self-Healing in Concrete. Construction and Building Materials 2014, 68, 110–119, doi:10.1016/j.conbuildmat.2014.06.018.

120. Wang, J.Y.; Soens, H.; Verstraete, W.; De Belie, N. Self-Healing Concrete by Use of Microencapsulated Bacterial Spores. Cement and Concrete Research 2014, 56, 139–152, doi:10.1016/j.cemconres.2013.11.009.

121. Wang, Y.; Geng, Y.; Ranzi, G.; Zhang, S. Time-Dependent Behaviour of Expansive Concrete-Filled Steel Tubular Columns. Journal of Constructional Steel Research 2011, 67, 471–483, doi:10.1016/j.jcsr.2010.09.007.

122. Wu, M.; Hu, X.; Hu, Z.; Zhao, Y.; Cheng, W.; Lu, W. Two-Component Polyurethane Healing System: Effect of Different Accelerators and Capsules on the Healing Efficiency of Dynamic Concrete Cracks. Construction and Building Materials 2019, 227, 116700, doi:10.1016/j.conbuildmat.2019.116700.

123. Wu, M.; Hu, X.; Zhang, Q.; Xue, D.; Zhao, Y. Growth Environment Optimization for Inducing Bacterial Mineralization and Its Application in Concrete Healing. Construction and Building Materials 2019, 209, 631–643, doi:10.1016/j.conbuildmat.2019.03.181.

124. Wu, M.; Johannesson, B.; Geiker, M. A Review: Self-Healing in Cementitious Materials and Engineered Cementitious Composite as a Self-Healing Material. Construction and Building Materials 2012, 28, 571–583, doi:10.1016/j.conbuildmat.2011.08.086.

125. Wu, Z.; Wong, H.S.; Buenfeld, N.R. Influence of Drying-Induced Microcracking and Related Size Effects on Mass Transport Properties of Concrete. Cement and Concrete Research 2015, 68, 35–48, doi:10.1016/j.cemconres.2014.10.018.

126. Xu, J.; Yao, W. Multiscale Mechanical Quantification of Self-Healing Concrete Incorporating Non-Ureolytic Bacteria-Based Healing Agent. Cement and Concrete Research 2014, 64, 1–10, doi:10.1016/j.cemconres.2014.06.003.

127. Yazıcı, H. The Effect of Silica Fume and High-Volume Class C Fly Ash on Mechanical Properties, Chloride Penetration and Freeze–Thaw Resistance of Self-Compacting Concrete. Construction and Building Materials 2008, 22, 456–462, doi:10.1016/j.conbuildmat.2007.01.002.

128. Yu, J.; Li, H.; Leung, C.K.Y.; Lin, X.; Lam, J.Y.K.; Sham, I.M.L.; Shih, K. Matrix Design for Waterproof Engineered Cementitious Composites (ECCs). Construction and Building Materials 2017, 139, 438–446, doi:10.1016/j.conbuildmat.2017.02.076.

129. Zemann, M.; Herrmann, N.; Dehn, F. Calcite Formation on Steamed Concrete Surfaces and Its Potential for Sealing Cracks. Construction and Building Materials 2019, 203, 1–8, doi:10.1016/j.conbuildmat.2019.01.091.

130. Zhang, J.; Liu, Y.; Feng, T.; Zhou, M.; Zhao, L.; Zhou, A.; Li, Z. Immobilizing Bacteria in Expanded Perlite for the Crack Self-Healing in Concrete. Construction and Building Materials 2017, 148, 610–617, doi:10.1016/j.conbuildmat.2017.05.021.

131. Zhang, J.; Zhao, C.; Zhou, A.; Yang, C.; Zhao, L.; Li, Z. Aragonite Formation Induced by Open Cultures of Microbial Consortia to Heal Cracks in Concrete: Insights into Healing Mechanisms and Crystal Polymorphs. Construction and Building Materials 2019, 224, 815–822, doi:10.1016/j.conbuildmat.2019.07.129.

132. Zhang, P.; Wittmann, F.H.; Lura, P.; Müller, H.S.; Han, S.; Zhao, T. Application of Neutron Imaging to Investigate Fundamental Aspects of Durability of Cement-Based Materials: A Review. Cement and Concrete Research 2018, 108, 152–166, doi:10.1016/j.cemconres.2018.03.003.

133. Zhang, Z.; Ding, Y.; Qian, S. Influence of Bacterial Incorporation on Mechanical Properties of Engineered Cementitious Composites (ECC). Construction and Building Materials 2019, 196, 195–203, doi:10.1016/j.conbuildmat.2018.11.089.

134. Zhu, Y.; Yang, Y.; Yao, Y. Autogenous Self-Healing of Engineered Cementitious Composites under Freeze–Thaw Cycles. Construction and Building Materials 2012, 34, 522–530, doi:10.1016/j.conbuildmat.2012.03.001.

135. Ziari, A.; Kianoush, M.R. Investigation of Direct Tension Cracking and Leakage in RC Elements. Engineering Structures 2009, 31, 466–474, doi:10.1016/j.engstruct.2008.09.011.

136. Ziari, A.; Reza Kianoush, M. Investigation of Flexural Cracking and Leakage in RC Liquid Containing Structures. Engineering Structures 2009, 31, 1056–1067, doi:10.1016/j.engstruct.2008.12.019.
